# The life cycle of the potentially zoonotic trematode *Metagonimus romanicus* (Digenea: Heterophyidae): New insights from published and original data

**DOI:** 10.1016/j.fawpar.2025.e00276

**Published:** 2025-07-05

**Authors:** Mikuláš Oros, Miroslava Soldánová, Daniel Barčák, Petra Kundid, Caroline Jepkorir Kibet, Roman Kuchta, Martina Orosová, Tomáš Scholz

**Affiliations:** a Institute of Parasitology, Slovak Academy of Sciences, Hlinkova 3, 040 01 Košice, Slovakia; b Institute of Parasitology, Biology Centre, Czech Academy of Sciences, Branišovská 31, 370 05 České Budějovice, Czech Republic; c Faculty of Science, University of South Bohemia in České Budějovice, Branišovská 31a, 370 05 České Budějovice, Czech Republic

**Keywords:** Cercariae, Metacercariae, Snails, Fish, Freshwater, Europe

## Abstract

Fish-borne zoonoses are emerging worldwide, and although most human cases remain confined to tropical regions, particularly Southeast and East Asia, a few cases have been reported in Europe. This review summarizes published and new data on the life cycle of *Metagonimus romanicus* (misidentified as *M. yokogawai*, a human pathogen common in East Asia), a heterophyid trematode and one of the potentially fish-borne parasites in Europe. *Metagonimus romanicus* is distributed from the middle Danube in Central Europe (Slovakia) to eastern Ukraine (including the rivers of the Black Sea basin). Its distribution area coincides with that of its first intermediate hosts, the melanopsid snails *Microcolpia daudebartii acicularis* and *Esperiana esperi*. While *M. romanicus* most likely has a strict specificity for its first snail hosts, metacercariae are generalists and have been detected in over 50 freshwater fish species from 18 families, especially leuciscids. Despite its wide distribution and frequent occurrence in freshwater fish, the zoonotic potential of *M. romanicus* appears to be low. This is probably due to the exclusive localization of the metacercariae in the fish scales rather than musculature, as well as the rare consumption of raw or undercooked fish in Europe. However, some risk remains and a small number of undiagnosed human infections due to accidental ingestion of scales during the preparation and cleaning of fish cannot be ruled out.

## Highlights

• The review contains published and original data on the life cycle of *Metagonimus romanicus* in Europe.
• *Microcolpia daudebartii acicularis* was confirmed as the first intermediate host in Europe.
• First molecular and morphological characterization of cercariae from snail hosts.
• Metacercariae of *M. romanicus* are found in the scales of a variety of freshwater fish.

## Graphical abstract

**Figure:**
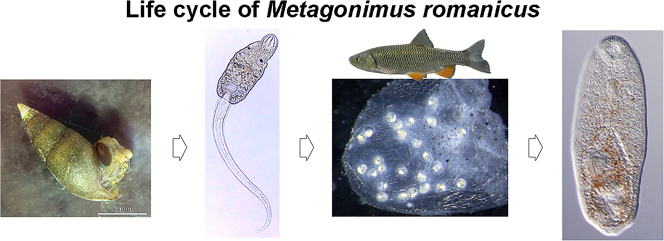

## 1 Introduction

Trematodes (Digenea) are widespread parasites of all major vertebrate groups, with several species being pathogenic to their hosts, including humans (Chai, 2019; Xiao et al., 2015). Trematodes of the family Heterophyidae use fish as second intermediate hosts and humans can become infected by eating raw or undercooked fish containing larvae of certain species, in particular those of the genera *Heterophyes* Cobbold, 1886, *Haplorchis* Looss, 1899 or *Metagonimus* Katsurada, 1912 (Chai, 2019). The species of the latter genus have been reported as common parasites of humans in East Asia (Chai et al., 2009), but the most common species, *M. yokogawai* (Katsurada, 1912), has also been reported in Europe (Chai, 2019). However, recent studies by Cech et al. (2023) and Scholz et al. (2024) provided evidence that this species does not occur in Europe, while *M. romanicus* (Ciurea, 1915) is probably the only member of the genus in European freshwater bodies.

*Metagonimus romanicus* was originally described as *Loossia romanica* in a domestic dog (*Canis familiaris*) from the Danube Delta in Romania (Ciurea, 1915). It is a widespread intestinal parasite in a variety of piscivorous birds and mammals in central and southeastern Europe, particularly in the lower reaches of rivers flowing into the Black Sea, with the most recent records coming from the middle and lower reaches of the Danube (Scholz et al., 2024). While data on the occurrence of metacercariae of *M. romanicus* (previously misidentified as *M. yokogawai*) in European freshwater fish are relatively common, information on the first intermediate hosts (snails) is limited to a few reports in which the trematode is also referred to as *M. yokogawai* or *Metagonimus* sp. (Arabuli et al., 2024; Gradowski, 1999; Iskova et al., 1995; Ivasiuk and Losev, 2019; Manafov, 2011; Olenev, 1979; Vojtek, 1974; Zdun, 1961).

As most trematodes, including heterophyids, are thought to exhibit narrow or strict specificity at the level of the first (snail) intermediate host (e.g., Faltýnková et al., 2016), the presence of snail hosts is crucial for a reliable assessment of the trematode life cycle and, consequently, the actual distribution and diversity of trematode species. In contrast, the low specificity of some trematodes for the second intermediate and definitive hosts allows the occurrence of trematodes outside the geographical range of their first snail intermediate hosts, i.e., in areas where the entire life cycle cannot be completed. From an epidemiological point of view, it is therefore important to map the occurrence of medically important trematodes in snail hosts, but also to reliably determine the range of their second intermediate hosts harboring metacercariae, the infective stages for definitive hosts, including humans.

In the absence of comprehensive epizootiological and epidemiological studies, snails and fish were studied to assess the distribution of *M. romanicus* along the middle reaches of the Danube. We summarize published and new data on the life cycle of *M. romanicus* (commonly misidentified as *M. yokogawai*, a human parasite common in East Asia (Chai, 2019)), including the first confirmed snail host of *M. romanicus* based on genetic data and detailed information on the host range of metacercariae from fish.

## 2 Materials and methods

### 2.1 Snail intermediate hosts

The snail collection was carried out in eastern and southern Slovakia during five sampling occasions between 2022 and 2024 (August 2022, June 2023, August 2023, April 2024 and June 2024). The snails (*n* = 6953) belonging to nine families: Bithyniidae (378 examined/31 infected), Lithoglyphidae (472/116), Lymnaeidae (2812/232), Melanopsidae (593/64), Neritidae (262/115), Physidae (1143/2), Planorbidae (1026/21), Valvatidae (4/0) and Viviparidae (263/55) were randomly hand-picked or collected with hand-nets from aquatic vegetation, stones and sediment along the shore of 51 localities covering diverse types of freshwater water bodies (reservoirs, ponds, channels, rivers and their blind arms in floodplains). In the laboratory, snails were first examined individually for the emergence of cercariae (patent infections) and then dissected by pressing their tissues between two glass slides to detect prepatent infections (intramolluscan stages, i.e., sporocysts and/or rediae) according to the standard procedure (e.g., Soldánová et al., 2010).

Cercariae were observed and identified live under a light microscope (Olympus BX51 with Nomarski interference contrast) using relevant keys and other sources (Faltýnková et al., 2007, Faltýnková et al., 2008). Pleurolophocercous cercariae isolated from snails of the genus *Microcolpia* (Caenogastropoda, Melanopsidae) and the gravel snail *Lithoglyphus naticoides* (Pfeiffer) (Caenogastropoda, Lithoglyphidae), which morphologically corresponded to heterophyid cercariae (profoundly long tail and pigmented eye-spots) were then separated and representative samples of cercariae and rediae were fixed in 96 % molecular ethanol for DNA sequencing. Cercariae were also fixed in cold (unheated) and hot 4 % formaldehyde solution (formalin) for morphometric analysis. Snails were measured with an electronic digital calliper with an accuracy of 0.5 mm.

### 2.2 Fish intermediate hosts

**Fig. 1:**
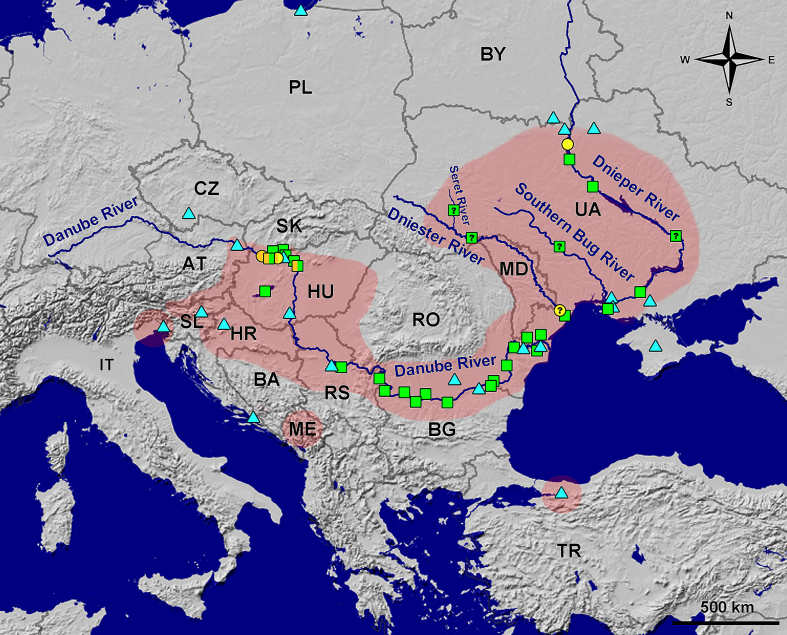
The map shows the occurrence of *Metagonimus romanicus* (Ciurea, 1915) in Europe in the stages cercariae (circle), metacercariae (square) and adults (triangle); positive sampling sites of this study are marked in orange, other colours represent literature data. Localities that are too close to each other are merged; a question mark means that the exact locality in the river was not mentioned in the original publication. The red area shows the distribution of suitable snail hosts of the genera *Microcolpia* and *Esperiana* according to various sources (see text). *Abbreviations*: AT, Austria; BA, Bosnia and Herzegovina; BG, Bulgaria; BY, Belarus; CZ, Czech Republic; HR, Croatia; HU, Hungary; IT, Italy; MD, Moldova; ME, Montenegro; PL, Poland; RO, Romania; RS, Republic of Serbia; SK, Slovakia; SL, Slovenia; TR, Turkey; UA, Ukraine. (For interpretation of the references to colour in this figure legend, the reader is referred to the web version of this article.)

Fish collections were carried out between 2022 and 2024 in the Austrian (Altenwörth), Slovakian (Veľký Lél) and Hungarian (near Szentendre) parts of the Danube River and in the mouth of the Morava and Dyje rivers (Brodské and Břeclav) in the Czech Republic (Fig. 1). Fish were caught by electrofishing under the permits Nos. 35/2023 and 54/2024 (Slovak Republic), NÖ LFV-E-46/202 (Austria) and MZE-68283/2022–16232 (Czech Republic). The study was approved by the Ethics Committee of the Institute of Parasitology of the Slovak Academy of Sciences (Košice, Slovakia) (approval No. 1/2020/PaU) and by ARRIVE guidelines (Percie du Sert et al., 2020).

A total of 26 fish species belonging to seven families (Supplementary Table S1) were examined for metacercariae of *M. romanicus* under a stereomicroscope (all organs with focus on the scales, fins and muscles were examined for parasites). The prevalence and intensity of infection were calculated; the latter was determined by counting metacercariae of *M. romanicus* in at least 50 positive scales. In case of low infection intensity (i.e., less than 50 positive scales), all scales were examined.

Some positive scales and fins were digested in an artificial gastric juice (1 g of pepsin (1: 10000) and 7 ml of concentrated hydrochloric acid in 1000 ml of 0.9 % sodium chloride) at 37 °C for 1 h to liberate encysted metacercariae from the scales. The cysts were then collected from the sediment and identified under a light microscope according to Vojtek (1974). Some of them were transferred to the excystation medium (2.5 g of pancreatin and 0.25 g NaHCO_3_ in 50 ml deionized water) at 37 °C until most of the metacercariae had hatched from the cysts (this usually took up to 30 min.) and then placed in 0.9 % saline solution (see Cech et al., 2023). Cysts were fixed in 96 % molecular ethanol; excysted metacercariae were fixed in hot saline and stored in 96 % molecular ethanol for subsequent morphological and molecular analyses.

### 2.3 Molecular study

Total genomic DNA was extracted from 16 individual cercariae (two per each of eight snail specimens), 15 metacercariae (at least one per fish species with sufficient number of metacercariae) and seven snail tissues using the Qiagen DNeasy Blood & Tissue kit (Qiagen, Hilden, Germany) according to the manufacturer's recommendations, but 40 μl of ddH_2_O was used for elution of parasite DNA.

Parasites were genotyped based on two nuclear markers; the D1-D3 region of the large ribosomal subunit (28S rRNA gene) was amplified with ZX1 and 1500R primers (Bray et al., 2009; Olson et al., 2003), and the region of internal transcribed spacers 1 and 2 separated by the 5.8S ribosomal subunit (here referred to as ITS) was amplified with forward (5′-GGAAGTAAAAGTCGTAACAAG-3′) and reverse (5′-GATATGCTTAARTTCAGCGGG-3′) primers, which are reverse and complementary versions of ZX1 and WormB (Bray et al., 2009; Littlewood and Olson, 2001). The snail hosts were genotyped based on partial gene for cytochrome *c* oxidase subunit I (COI), which was amplified with the universal primers LCO 1490 and HCO 02198 (Folmer et al., 1994).

Amplification was performed with Takara Ex Taq polymerase (Takara Co., Tokyo, Japan); cycling conditions were as follows: initial denaturation at 95 °C for 3 min., 35 cycles of denaturation at 95 °C for 30 s., annealing at 56 °C (28S, ITS) or 50 °C (COI) for 30 s. and elongation at 72 °C for 1 min.; final termination was held at 72 °C for 5 mins. The PCR products were verified on a 1 % agarose gel and enzymatically purified (Werle et al., 1994). Sanger sequencing was performed with PCR primers; the templates of the parasites were additionally sequenced with the internal primers 300R and 900F (28S) as well as HO1850r (5′-CAGCACATCCCGTCAC-3´) and Tr2120f (5´-CGATGAAGAGTGCAGCYAAC-3′) for ITS. At least two raw reads were *de-novo* aligned to create contiguous sequences that were manually inspected for ambiguous positions. The newly generated sequences were deposited in the GenBank database.

The snails were identified by comparison of a representative, partial COI sequence with available data using BLAST with default settings. Representative parasite sequences were included in the phylogenetic analysis comprising isolates of the genus *Metagonimus* (see Supplementary Table S2). The alignments of the three markers (18S, 28S and ITS) were constructed separately in MAFFT v7.490 with algorithms *E*-INS-i (Katoh and Standley, 2013). The length of the trimmed alignments was 1832 bp (18S), 1245 bp (28S) and 1137 bp (ITS), totaling 4214 bp. The optimal nucleotide substitution models HKY + F (18S), TVM + F + I + G4 (28S) and TVM + F + G4 (ITS) were calculated in ModelFinder (Chernomor et al., 2016; Kalyaanamoorthy et al., 2017) using the AICc criterion. Phylogenetic analysis was performed on a concatenated alignment of the three genetic markers mentioned above with IQtree 2.0.5. using ultrafast bootstrapping of 1000 replicates (Hoang et al., 2018; Minh et al., 2020).

### 2.4 Morphological study

Live and fixed larvae (cercariae, rediae, metacercariae) were observed and photographed by Olympus BX51 light microscope with the Promicam 3-5CP digital camera and analyzed in the program ImageJ v.1.53e (Abràmoff et al., 2004). Selected specimens were prepared for scanning electron microscopy (SEM) following the procedure of Chervy (2024) and photodocumented by JEOL JSM-7401 F field emission electron microscope (Biology Centre of the Czech Academy of Sciences, Czech Republic).

The dimensions (in micrometers) of live cercariae (*n* = 29) and formalin-fixed cercariae (*n* = 93) are based on 18 and 10 morphometric parameters, respectively. In addition, two ratios were calculated from the measurements depending on the fixation method (Table 1, Supplementary Fig. S1). The measurements of rediae (*n* = 10) are based on live specimens. Microscopic images of the larval stages from all fixatives were taken from temporary slides using the proportionate amount of liquid to avoid excessive floating or flattening. Live cercariae were photographed several times in succession to capture both the extended and contracted positions, and values were averaged to minimise possible measurement bias. The width values correspond to the maximum width measurements. The measurements obtained in our study were compared with the only available dimensions of cercariae of *Metagonimus* spp. reported from Europe, including the Transcaucasian countries as geographical regions extending their territory into Europe (Manafov, 2011; Olenev, 1979; Zdun, 1961) (see Appendix A Supplementary data and Supplementary Table S3).

**Table 1:** Measurements of cercariae of *Metagonimus romanicus* (Ciurea, 1915) isolated from the snail host *Microcolpia daudebartii acicularis* (Férussac). Data (in micrometers) are presented as mean ± SD followed by a range in parentheses. See Supplementary Fig. S1 for an illustration and abbreviations of the morphometric features.

Fixation method	Live	Hot formalin	Cold formalin
*N* cercariae	29	48	45
Total length	634 ± 71 (506–848)	561 ± 46 (480–654)	561 ± 29 (501–621)
Body length (BL)	190 ± 36 (142–325)	160 ± 17 (141–202)	138 ± 11 (118–175)
Body width (BW)	110 ± 13 (88–137)	75 ± 9 (50–95)	85 ± 8 (68–100)
Tail length (TL)	444 ± 43 (334–523)	401 ± 35 (337–469)	423 ± 28 (364–488)
Tail with tegument width (TWte)	36 ± 5 (24–49)	–	–
Tail without tegument width (TW)	29 ± 5 (23–41)	28 ± 3 (22–36)	29 ± 4 (21–39)
Tegument width (teW)	5 ± 1 (4–7)	–	–
Tail socket length (TSL)	22 ± 3 (18–32)	15 ± 2 (10−20)	16 ± 3 (12–26)
Dorsal fin-fold width (DffW)	23 ± 2 (21–27)	–	–
Dorsal fin-fold – start from tail socket (Dff(TS))	79 ± 11 (53–95)	–	–
Oral sucker length (OSL)	46 ± 4 (37–55)	36 ± 2 (30–41)	37 ± 3 (26–42)
Oral sucker width (OSW)	36 ± 6 (28–47)	29 ± 4 (23–46)	30 ± 3 (23–36)
Pharynx length (PL)	13 ± 2 (10–15)	–	–
Pharynx width (PW)	12 ± 2 (8–17)	–	–
Eye-spots to anterior body end (ESbe)	73 ± 16 (41–111)	46 ± 7 (33–66)	35 ± 5 (23–47)
Eye-spots diameter (ESd)	9 ± 2 (7–13)	7 ± 1 (5–9)	8 ± 1 (6–9)
Ventral sucker length (VSL)	22 ± 3 (18–27)	–	–
Ventral sucker width (VSW)	23 ± 3 (20–28)	–	–
Tail length/body length (ratio)	2.4 ± 0.3 (1.6–3.1)	2.5 ± 0.2 (2.1–3.0)	3.1 ± 0.3 (2.3–3.7)
Oral/ventral sucker width (ratio)	1.5 ± 0.4 (0.9–1.9)	–	–

A multivariate analysis of variance (MANOVA) was first carried out to statistically assess the effects of the three fixation methods on cercarial morphometry. Following a significant result, a series of univariate (one-way) ANOVAs were performed for each morphometric parameter, followed by Tukey's (HSD) *post-hoc* tests to determine specific group differences. Data consisting of nine morphometric features (BL, BW, TL, TW, TSL, OSL, OSW, ESbe and ESd; see abbreviations in Table 1 and Supplementary Fig. S1) were analyzed for each cercariae group (29 live, 48 hot formalin-fixed and 45 cold formalin-fixed cercariae). All data were ln-transformed to normalise their distribution. Analyses were performed using the Statistica 7.0 software package (StatSoft Inc., Tulsa, OK, USA), with the significance level set at 0.05.

To further investigate the relationship between the morphometric parameters and the fixation methods, principal component analysis (PCA) and linear discriminant analysis (LDA) were performed on the same dataset of cercariae groups. PCA was used to visualise variance patterns and clustering of groups, while LDA was used to assess group separation and the contribution of each parameter to the discrimination between fixation methods.

The encysted metacercariae (cysts) were measured either alive (within one hour of their isolation from the scales) or after fixation in 96 % ethanol to evaluate the effect of the ethanol on cyst size. The excysted metacercariae, which were fixed in hot saline and stored in 96 % ethanol, were stained with Mayer's carmine, destained in acid ethanol, dehydrated in an ethanol series, clarified with clove oil, embedded in Canada balsam and measured as permanent preparations (see Scholz et al., 2024).

Morphological vouchers of cercariae and metacercariae were deposited in the Helminthological collection of the Institute of Parasitology, Biology Centre of the Czech Academy of Sciences in České Budějovice, Czech Republic (Coll. No. IPCAS D-876).

## 3 Results

### 3.1 Molecular identification

**Fig. 2:**
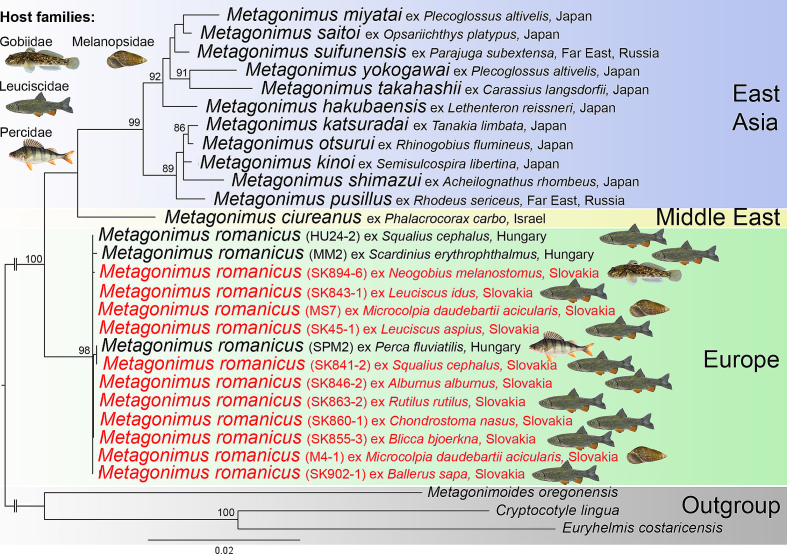
Phylogenetic position of *Metagonimus romanicus* (Ciurea, 1915) within its congeneric species. Note that all isolates of *M. romanicus*, including newly generated (red) from one snail species and nine fish species from two families, are genetically almost identical in terms of the genetic markers used. The interrelationships are depicted as a consensus tree generated by ultrafast bootstrap approximation approach based on a concatenated dataset of three nuclear genetic markers (18S rRNA and, 28S rRNA, and ITS region). Only nodal supports above 80 are shown and the scale bar represents the number of substitutions per site. (For interpretation of the references to colour in this figure legend, the reader is referred to the web version of this article.)

Genotyping of cercariae and metacercariae confirmed that one snail species and at least nine of the 26 fish species examined (metacercariae were not genetically confirmed from all fish species) serve as suitable intermediate hosts for *M. romanicus* in the Slovakian and Hungarian parts of the Danube (Fig. 2). Pairwise comparison using two molecular markers (1336 bp long 28S and 1208 bp long ITS) involved 31 specimens sequenced in this study and revealed very low genetic diversity between them (0–0.3 % for 28S and 0–0.1 % for ITS). The diversity was mainly caused by few ambiguities repeatedly detected at specific positions in the sequences of most of the specimens and therefore probably reflects the intraindividual variability of these multicopy genetic markers. Due to the low genetic diversity of the newly generated sequences, only two representative isolates of cercariae (one from each positive locality) and nine isolates of metacercariae (one from each fish host species) were included in the phylogenetic analysis.

In the phylogenetic tree, the new isolates formed a strongly supported clade with *M. romanicus* specimens collected in Hungary (Cech et al., 2023; Scholz et al., 2024) (Fig. 2). Within the genus *Metagonimus*, *M. romanicus* formed the earliest diverging branch as a sister group to *Metagonimus ciureanus* (Witenberg, 1929) and all species from Asia (Scholz et al., 2024).

Genotyping of seven infected snails from two sampling sites in the Slovakian Danube (two snails from Gabčíkovo and five snails from Veľký Lél) revealed seven identical sequences of the partial COI gene. Of these, one representative sequence (657 bp long) was selected for BLAST comparison, and the isolates of *Microcolpia daudebartii acicularis* (Férussac) (acc. Nos. MK094109 from Slovenia (Neiber and Glaubrecht, 2019) and KJ831012, KJ831014, KJ831015, KJ831036 of unknown origin) were identified as the five best matches. The pairwise diversity between these six sequences was 0.2–0.3 %, which corresponds to expected intraspecific variability.

The newly generated sequences were deposited in GenBank (*M. romanicus*: PV777974–83 and PV779653–63, see Supplementary Table S2; *M. daudebartii acicularis*: PV777662).

### 3.2 First snail intermediate hosts

Of all the snails collected and examined, *Microcolpia daudebartii acicularis* (Férussac) (Melanopsidae) was the only gastropod infected with *Metagonimus romanicus* (Supplementary Fig. S2). A total of 593 *M*. *daudebartii acicularis* were collected from three localities in southern Slovakia. Overall, 10.8 % of the snails were infected with different trematode species, but only 7.6 % (*n* = 45) were infected with *M*. *romanicus* larvae at two localities: (i) Bočianské arm in the Danube floodplains near the town of Gabčíkovo (47°53′25.74″N, 17°30′51.88″E) in June 2023 (*n* = 167; 3.6 %) and June 2024 (*n* = 304; 11.2 %), and (ii) at Danube River near Veľký Lél Island (47°45′3.824″N, 17°57′8.08″E) in April 2024 (*n* = 32; 15.6 %) (Fig. 1). The third locality at the Danube near Veľký Lél Island (47°44′32.28″N, 17°54′0.72″E), which was sampled in June 2023, was free of infections (*n* = 90). The overall size of *M. daudebartii acicularis* (shell length × width, with mean ± SD in mm) was 16.1 ± 2.2 × 6.4 ± 0.9, but snails infected with *M*. *romanicus* were generally larger (20.6 ± 2.2 × 8.0 ± 0.9).

### 3.3 Description of cercariae (Fig. 3, Fig. 4; Table 1)

**Fig. 3:**
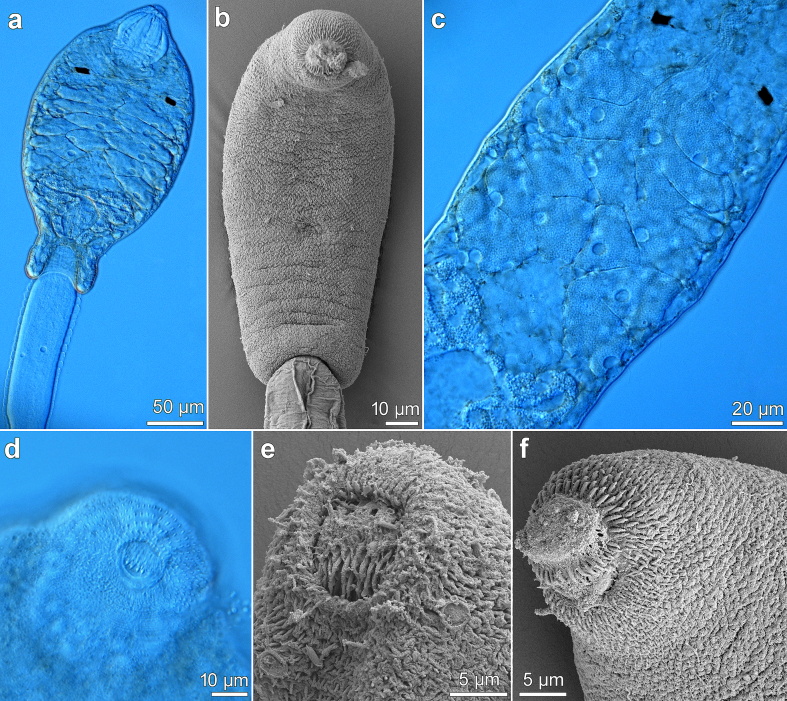
Cercaria of *Metagonimus romanicus* (Ciurea, 1915) from *Microcolpia daudebartii acicularis* (Férussac), Danube, Slovakia (light and scanning electron microscopy, SEM). (a) general view of body, ventral (b) general view of body, ventral (SEM) (c) penetration gland-cells (d) oral spines with four large spines in first row, ventral (e) oral spines (SEM), ventral (f) everted oral sucker with spines, ventrolateral (SEM).

**Fig. 4:**
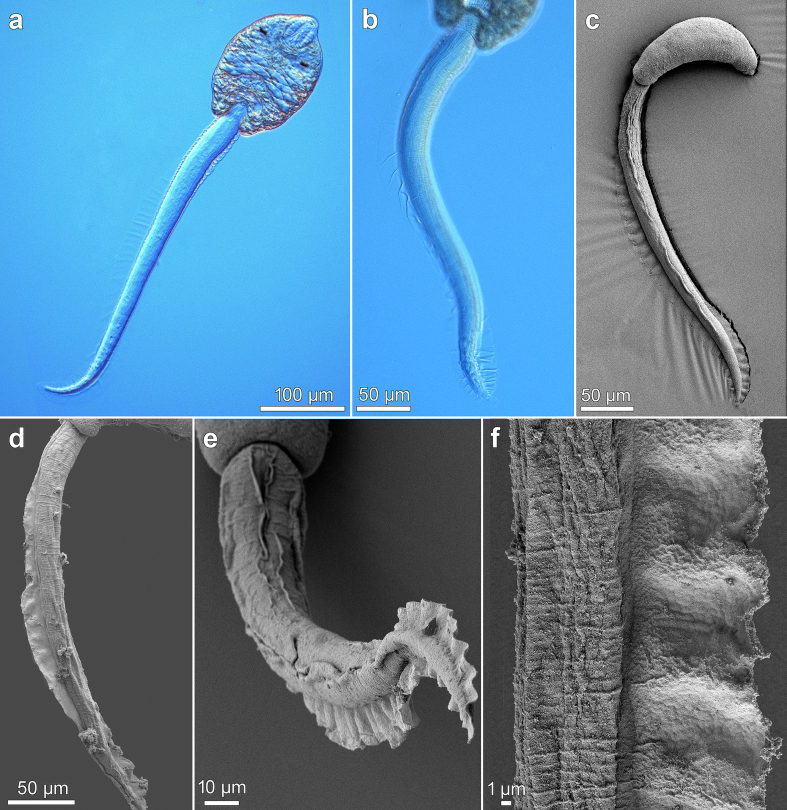
Cercaria of *Metagonimus romanicus* (Ciurea, 1915) from *Microcolpia daudebartii acicularis* (Férussac), Danube, Slovakia (light and scanning electron microscopy, SEM). (a) general view, ventral (b) tail with fin-fold in contracted position (c) general view, dorsolateral (SEM) (d) tail with a dorsal fin-fold (SEM) (e) tail tip with ventral fin-fold and part of dorsal fin-fold (SEM) (f) detail of dorsal fin-fold (SEM).

Live specimens (*n* = 29; Table 1): Cercaria pleurolophocercous. Total length (sum of body and tail length) up to 850 μm. Body elongate-oval, with brown pigment (Figs. 3a; 4a), covered with fine spines (Fig. 3b), larger spines present in anterior body end (Fig. 3f). Two pigmented eye-spots conspicuous, usually rectangular (Fig. 3a, c) or X-shaped (occasionally oval-shaped). Pharynx spherical, immediately posterior to eye-spots. Prepharynx short, oesophagus and caeca not observed. Penetration gland-cells large, in two groups of seven on each side of body (14 in total), extending across midbody in longitudinal series posterior to eye-spots, with last pair equatorially posterolateral to ventral sucker (Fig. 3c). Duct outlets of penetration glands widen and open in 4 groups at anterior tip of body (Fig. 3a). Oral sucker conspicuous, subspherical (slightly longer than wide) (Fig. 3a), width exceeds width of ventral sucker (ratio about 1.5); oral spines in 2–3 transverse rows with 4 large spines in first row, followed by 10–12 smaller spines (Fig. 3d, e). Ventral sucker weakly developed (observable only in 7 specimens), but distinctly smaller than oral sucker, spherical. Genital primordium elliptical, posterior to ventral sucker. Cystogenous glands on both sides of body, extending from anterior to posterior body end. Excretory bladder Y-shaped, thick-walled (Fig. 3a). Flame-cell formula not identified.

Tail long and slender, approximately 2.5 × longer than body (ratio 2.4) (Fig. 4a–c), inserted in tail socket (Figs. 3a; 4b), spineless (Fig. 4d–f). Tail equipped with pronounced dorsoventral undulating fin-fold (Fig. 4a–e) and tegumental membrane, most prominent anteriorly and gradually tapering towards half of tail (Figs. 3a; 4a); tail reaching its maximum width shortly posterior to tail socket (Figs. 3a; 4a, b). Dorsal fin-fold covers 3/4 of tail length (Fig. 4a–c), appearing at a distance from tail base in socket and terminating at tail tip (Fig. 4a, d), gradually increasing in width and reaching its maximum about halfway down tail (Fig. 4f). Ventral fin-fold covers 1/4 of tail length, emerging at posterior part of tail and partially overlapping terminal part of dorsal fin-fold (Fig. 4e). Both fin-folds expand to surround tip of tail (Fig. 4e).

Cercariae fixed in hot (*n* = 48) and cold formalin (*n* = 45) of identical morphology, but significantly smaller than live specimens (see Table 1 and statistics below).

A comparative overview of the morphological and metrical differences to earlier records of *Metagonimus* cercariae is provided in Supplementary Table S3 and the corresponding remarks in Appendix A Supplementary data.

### 3.4 Description of rediae (Fig. 5)

**Fig. 5:**
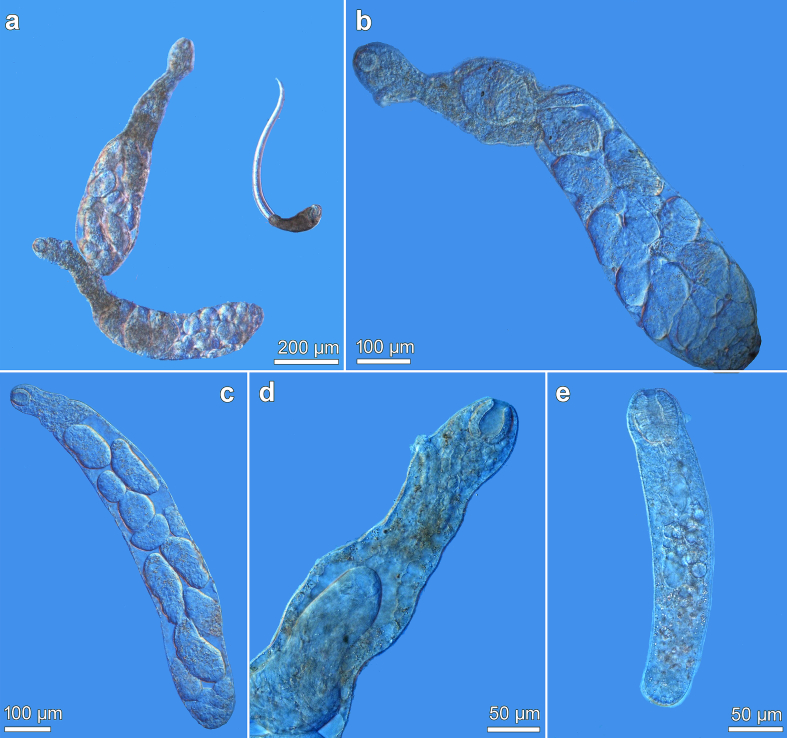
Rediae of *Metagonimus romanicus* (Ciurea, 1915) from *Microcolpia daudebartii acicularis* (Férussac), Danube, Slovakia; different developmental stages. (a) rediae in comparison to mature cercaria (b) redia (tapered anteriorly and widened posteriorly) containing germ cells and few cercariae in advanced stage of development (c) redia (tapered anteriorly) with germ cells (d) anterior end with pharynx (e) young redia with undifferentiated content.

The morphology and measurements of large daughter rediae, which contained either fully developed or immature cercariae, were as follows: live specimens (*n* = 10; mean ± SD in μm, followed by a range in parentheses): body cylindrical, 949 ± 134 long (748–1130) (Fig. 5a–c), anteriorly narrowed in pharyngeal region 72 ± 9 (63–88) (Fig. 5a–c), but often widened posteriorly 167 ± 27 (142–215) (Fig. 5b, c). Pharynx slightly subspherical, 41 ± 3 × 39 ± 1 (38–45 × 38–40) (Fig. 5d). Birth pore lateral at anterior tip of body, posterior to pharynx. Intestine short and narrow (Fig. 5c, e). Young redia (*n* = 1): Body 316 × 60, pharynx 53 × 43 (Fig. 5e).

Comparative remarks on the morphology of rediae based on previous studies are provided in Appendix A Supplementary data.

### 3.5 Statistical morphometric analysis of cercariae

The MANOVA analysis performed for nine morphometric parameters of cercariae (Supplementary Fig. S1a) revealed significant differences between the three fixation methods (Wilks' Lambda = 0.052, F_(18,178)_ = 33.31, *p* < 0.001), indicating considerable morphological differences between live specimens and those fixed in hot and cold formalin (Supplementary Fig. S1b–d).

**Fig. 6:**
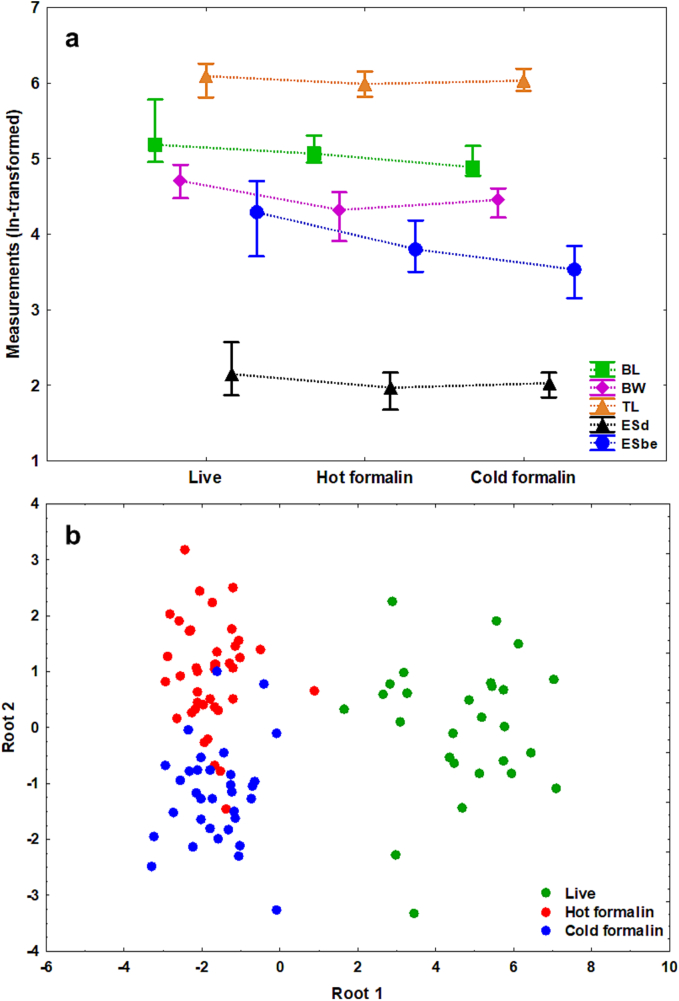
Specimens of cercariae of *Metagonimus romanicus* (Ciurea, 1915) showing results of (a) one-way ANOVA analyses showing differences in the main morphometric parameters across the three fixation methods (live cercariae, cercariae fixed in hot and cold formalin). Symbols denote the median values (50th percentile) and vertical bars non-outlier range referring to the smallest and largest values within 1.5 times the interquartile range. (b) Linear discriminant analysis (LDA) plot demonstrating clear separation of cercariae in relation to the three fixation methods.

The results of one-way ANOVA tests showed a strong influence of fixation methods on most morphometric traits (only tail width was not significant) (Supplementary Table S4). *Post-hoc* tests (Tukey's HSD) further revealed the specific nature of these differences with significantly larger live cercariae than those fixed in hot and cold formalin (Table 1; Fig. 6a; Supplementary Table S4). However, cercariae fixed in formalin showed different results between the two groups. For example, the body length was significantly reduced in cold formalin compared to hot formalin, but the body width showed an opposite trend. The tail was significantly shorter in hot formalin, but the effect of cold formalin was minimal, as the dimensions were comparable to those of live cercariae (albeit even smaller). In general, both hot and cold formalin resulted in significant morphometric changes, but with different effects on certain characteristics. In hot formalin, the dimensions of body length and the distance of the eye-spots to the anterior body end were larger (i.e., closer to live cercariae), whereas in cold formalin, larger dimensions were observed for body width, tail length and eye-spot diameter (Fig. 6a; Supplementary Table S4). The other parameters, such as the length of the tail socket and the dimensions of the oral sucker, were similar in both formalin fixatives (Supplementary Table S4).

Principal component (PCA) and linear discriminant (LDA) analyses emphasized the primary contribution of morphometric features of the cercariae to discriminate between fixation methods. PCA analysis revealed that the first two principal components explained 63.2 % of the variation in the dataset, with six morphometric traits making the largest contribution to the total variance. Body length and width, oral sucker length, eye-spot diameter and distance of eye-spots to anterior body end had the highest coefficients for the first component and explained more than half of the variance (PC1, 50.8 %), while tail width was the main contributor to the second principal component and accounted for additional variability (PC2, 12.4 %). The PCA scores plot (Supplementary Fig. S3) showed distinct clusters along the first axis corresponding to the three fixation methods, suggesting that the samples differed significantly in their morphometric profiles, especially for traits related to body size.

LDA analysis supported the PCA results and showed a clear separation between the three fixation methods (Wilks' Lambda = 0.058, F_(18,178)_ = 33.11, p < 0.001) (Fig. 6b). Live cercariae formed a distinct group, while specimens fixed in hot and cold formalin had overlapping but distinct groups (Fig. 6b). Discriminatory power was associated with four morphometric traits, body width, distance of eye-spots to anterior body end, tail socket length and tail width, which together contributed most to separation along the first discriminant axis (90.5 % of the variance). The second discriminant axis captured additional variance (9.5 %) and separated the groups mainly on the basis of body length and tail length.

Overall, these results demonstrate that fixation methods have a significant effect on key morphological characteristics of cercariae, with body width and distance of eye-spots to anterior body end (although correlated) being the most sensitive to formalin fixation, as these soft-bodied larvae shrink and deform significantly.

### 3.6 Second fish intermediate hosts

In the middle Danube in Slovakia, Austria and Hungary and in two rivers in the Czech Republic (lower Morava and Dyje), a total of 179 fish individuals belonging to 26 fish species from seven families (mainly Leuciscidae) were collected and examined between 2022 and 2024 (Fig. 1; Supplementary Table S1). Of these, only 12 species (46 % of all species examined) in Slovakia and Hungary harbored metacercariae of *M. romanicus* with an overall prevalence of 77 % (Table 2), while all 73 fish of nine species from Austria and the Czech Republic were negative (Supplementary Table S1).

**Table 2:** Infection parameters for metacercariae of *Metagonimus romanicus* (Ciurea, 1915) found within scales of fish collected in the Danube River in Slovakia and Hungary (positive localities). The infection intensity/fish is expressed as the number of metacercariae in each fish species, followed by the number of fish individuals examined in parentheses, and the infection intensity/scale indicates the average number of metacercariae per scale in each fish species, followed by a range in parentheses. The values were calculated based on the examination of up to 50 positive scales per fish individual.

Country	Slovakia and Hungary				
Fish host	No. examined	No. infected	Prevalence (%)	Infection intensity/fish	Infection intensity/scale
*Abramis brama*	2	1	50	34 (1)	1.00
*Alburnus alburnus*	8	7	88	14–77 (6)	1.30 (1–4)
*Ballerus sapa*	2	2	100	10–46 (2)	1.10 (1–3)
*Blicca bjoerkna*	2	1	50	96 (1)	1.90 (1–5)
*Chondrostoma nasus*	16	15	94	3–223 (7)	2.90 (1−12)
*Leuciscus aspius*	2	1	50	12 (1)	1.00
*Leuciscus idus*	7	6	86	112–387 (2)	5.90 (1–24)
*Neogobius melanostomus*	2	1	50	56 (1)	1.10 (1–2)
*Perca fluviatilis*	6	1	17	43 (1)	1.02 (1–2)
*Rutilus rutilus*	5	5	100	59–96 (3)	1.70 (1–9)
*Scardinius erythrophthalmus*	5	1	20	31 (1)	1.00 (1–2)
*Squalius cephalus*	26	23	88	15–366 (10)	3.00 (1–42)
**Total**	**83**	**64**	**77**	**3–387 (35)**	**2.40 (1–42)**

**Fig. 7:**
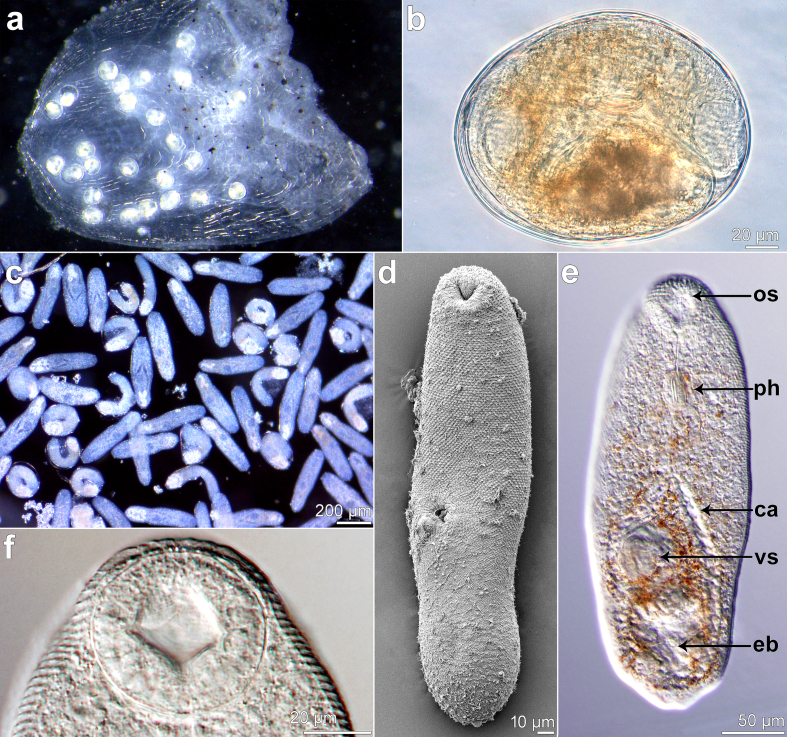
Metacercariae of *Metagonimus romanicus* (Ciurea, 1915) from cyprinoid fish, Danube, Hungary (light and scanning electron microscopy, SEM). (a) encysted metacercariae in fish scale (b) encysted metacercaria (c) excysted metacercariae (d) general view, ventral (SEM) (e) general view, ventral; os, oral sucker; ph, pharynx; ca, caeca; vs, ventral sucker; eb, excretory bladder (f) oral sucker, ventral.

The cysts with metacercariae of *M. romanicus* were located exclusively within the scales (on the underside of the scales) of the entire body of the infected fish (Fig. 7a). The epidemiological parameters are shown in Table 2, where chub (*Squalius cephalus* [L.]), ide (*Leuciscus idus* [L.]), white bream (*Blicca bjoerkna* [L.]) and nase (*Chondrostoma nasus* [L.]) were the most suitable hosts in terms of high infection intensity and prevalence. The intensity of infection was on average 2.4 metacercariae per scale (reaching up to 5.9), and one scale could be infected with up to 42 metacercariae (Table 2). The highest number of metacercariae detected in a single fish individual was 387 in ide (Table 2).

### 3.7 Description of metacercariae (Fig. 7)

Cyst (live, *n* = 4; ethanol-fixed, *n* = 8; mean ± SD in μm, followed by a range in parentheses): both live and ethanol-fixed cysts spherical or slightly elliptical, thin-walled; live cysts 206 ± 11 (191–216) long and 169 ± 11 (157–183) wide, cyst wall thickness 2.5 ± 0.6 (2–3) (Fig. 7a, b); ethanol-fixed cysts 172 ± 14 (151–187) long and 133 ± 15 (125–158) wide, cyst wall thickness 4.5 ± 0.5 (4–5).

Excysted metacercaria (fixed in hot saline and stored in 96 % ethanol; *n* = 8): body elongate, 315 ± 20 (289–387) long and 102 ± 14 (83–115) wide, tapering slightly posteriorly (Fig. 7c–e). Tegument entirely spinose; larger spines on anterior half of body (Fig. 7d–f). Oral sucker subventral, 45 ± 4 (41–52) × 48 ± 4 (43–55) (Fig. 7f). Prepharynx short, 19 ± 3 (14–27) in length, pharynx 28 ± 3 (25–33) × 25 ± 3 (19–29), gland cells present on both sides of pharynx. Oesophagus 41 ± 6 (36–53) long, bifurcating in middle of anterior-posterior axis of body (Fig. 7e). Ventral sucker excentric, 28 ± 4 (24–32) × 27 ± 2 (24–29), located 176 ± 8 (162–187) from anterior end of body (Fig. 7e). Primordial testes situated close to each other, diagonal position, excretory bladder Y-shaped (Fig. 7e).

Comparative remarks on the morphology of metacercariae and the spectrum of fish hosts based on previous studies are provided in Supplementary Table S5 and Appendix A Supplementary data.

## 4 Discussion

The present study provides the detailed molecular and morphological characterization of larval stages (cercariae and metacercariae) of the potentially zoonotic trematode *Metagonimus romanicus* in natural populations of snails and fish in Europe. The adults obtained from experimental infections have recently been described (Scholz et al., 2024), allowing us to reconstruct the life cycle of this trematode. *Metagonimus romanicus* has been misidentified in almost all previous studies as *M. yokogawai*, a species that commonly infects humans in East Asia (Chai and Jung, 2024), but is often erroneously reported from Europe, where only *M. romanicus* is found (Scholz et al., 2024).

Reports of *Metagonimus* cercariae in freshwater snails in Europe, including Transcaucasia, are scarce and limited to Slovakia (Vojtek, 1974), Ukraine (Gradowski, 1999; Iskova et al., 1995; Ivasiuk and Losev, 2019; Zdun, 1961), Georgia (Arabuli et al., 2024; Olenev, 1979) and Azerbaijan (Manafov, 2011). These cercariae were previously classified as *M. yokogawai* or *Metagonimus* sp. without considering the host specificity of the trematodes or the geographical distribution of the snail hosts. The sparse and inconsistent morphological descriptions (see Supplementary Table S3) have probably also contributed to the general assumption that cercariae found in Europe are identical to the Asian *M. yokogawai*.

In East Asia, the most common first intermediate hosts of *Metagonimus* species are the snails of the family Semisulcospiridae (Caenogastropoda), especially species of the genus *Semisulcospira* Boettger, which are common in Japan, China, Korea and Taiwan (e.g., Davis, 1969; Chai et al., 2005). In the Far East of Russia, snails of the genus *Juga* Adams et Adams (used in the literature as a synonym of *Parajuga* Prozorova et Starobogatov (Kantor et al., 2010; Strong and Köhler, 2009)) also serve as hosts (e.g., Besprozvannykh, 1989, Besprozvannykh, 2000). However, genetic and morphological data on different life stages, including adults, have shown that the trematodes in this area represent separate species, namely *Metagonimus suifunensis* Shumenko, Tatonova et Besprozvannykh, 2017 and *Metagonimus pusillus* Tatonova, Shumenko et Besprozvannykh, 2018 (Shumenko et al., 2017; Tatonova et al., 2013).

The occurrence of *Metagonimus* species in Europe and Transcaucasia seems to depend on the distribution of melanopsid snails, especially *Esperiana esperi* (Férussac), *Melanopsis praemorsa* (probably *M*. *mingrelica*, confirmed from Georgia and Azerbaijan (Bikashvili et al., 2025)) and *Microcolpia daudebartii acicularis* (syn. *Fagotia acicularis* and *Esperiana acicularis* (Glöer, 2019)) (Arabuli et al., 2024; Gradowski, 1999; Iskova et al., 1995; Ivasiuk and Losev, 2019; Manafov, 2011; Olenev, 1979; Vojtek, 1974; Zdun, 1961). However, the systematics of melanopsids is not well resolved as it is mainly based on shell morphology (Shumenko et al., 2017), which not only calls into question the species delimitation (Bikashvili et al., 2025; Neiber and Glaubrecht, 2019), but also the accuracy of reported geographic distributions.

Although the first evidence of the complete life cycle of *M*. *romanicus* (as *M. yokogawai*) probably comes from natural and experimental infections of hosts in Slovakia, it was never properly described (Vojtek, 1974). In our study, *M*. *daudebartii acicularis* was confirmed as the first intermediate host of *M*. *romanicus* based on the match with the partial COI sequence (Neiber and Glaubrecht, 2019). Together with molecular confirmation in several fish species, our study is the first to provide integrated molecular, morphological and morphometric data for larval stages in both intermediate hosts in Europe, thus completing the life cycle with the previously described adults and metacercariae (Cech et al., 2023; Ciurea, 1933a; Scholz et al., 2024). However, in addition to *M*. *daudebartii acicularis*, the presence of *M*. *romanicus* in *E*. *esperi* is also very likely, mainly due to the earlier record of the parasite (Zdun, 1961) and the overlapping occurrence and relatively wide distribution of both snail species in Central and Southeastern Europe, covering the Danube basin from Austria to the Danube delta (Tomović et al., 2014) and Ukraine (Dniester and Dnieper rivers) (Tytar and Makarova, 2015), including Slovakia (Čejka, 2011; Horsák et al., 2013; Ložek, 1956), and therefore requires further investigation.

The distribution of *M*. *romanicus* in Europe closely follows the distribution of snail hosts (Fig. 1) and is supported by numerous records of adults in bird and mammalian definitive hosts and of metacercariae in fish intermediate hosts (Cech et al., 2023; Scholz et al., 2024). The sporadic occurrence of adult *M*. *romanicus* in the Czech Republic (Sitko et al., 2006), Poland (Kanarek and Zaleśny, 2014) and Italy (Leonov, 1960) probably reflects the mobility of the definitive hosts (i.e., birds and mammals) rather than local transmission, as no suitable snail hosts are available in these countries (Scholz et al., 2024; Fig. 1).

Further evidence for the limited distribution of *M*. *romanicus* in Central and Southeastern Europe are records of *M*. ‘*yokogawai*’ and *Metagonimus* sp. in *M*. *praemorsa* snails in Georgia and Azerbaijan (Manafov, 2011; Olenev, 1979). While *M*. *romanicus* was confirmed molecularly in *M*. *daudebartii acicularis* (present study) and probably also infects *E*. *esperi*, the exact identity of *Metagonimus* cercariae reported from Transcaucasia remains uncertain, mainly due to differences in snail distribution and the morphology of cercariae. The distribution of *M*. *daudebartii acicularis* and *E*. *esperi* is Pontic and includes the rivers of the Black Sea region in Eastern Europe up to the Danube valley in Hungary and Slovakia (Glöer, 2019). In contrast, *M*. *praemorsa* is considered a western Mediterranean species that occurs mainly in southern Spain and North Africa (Glöer, 2019; Neiber and Glaubrecht, 2019) and may therefore have been misidentified due to the similar shape and high shell plasticity typical of *Melanopsis* species (Glöer, 2019).

Several conspecifics such as *Melanopsis* *ammonis* Tristram, 1865, *M*. *buccinoidea* (Olivier, 1801), *M. costata* (Olivier, 1804), *M*. *mingrelica* and *M. saulcyi* Bourguignat, 1853 occur in Georgia and Azerbaijan or neighbouring countries and could therefore represent the actual snail hosts (Bikashvili et al., 2025; Glöer, 2019; Kantor et al., 2010; Neiber and Glaubrecht, 2019). Furthermore, based on the available morphological data, it is likely that the cercariae found in Transcaucasia represent at least two different species of *Metagonimus*, one of which could be *M*. *ciureanus*. Adult specimens of this parasite have been documented in Georgia (Dzhikia, 1934; Kozlov, 1977; Rodonaya, 1967), Israel (type locality) (Dzikowski et al., 2004; Witenberg, 1929) and Jordan (Ismail et al., 1983) and may also occur in other neighbouring Middle Eastern countries such as Abkhazia, Armenia, Azerbaijan, Iran, Iraq and probably also in southwestern Russia (Scholz et al., 2024).

Previous records of cercariae of *Metagonimus* spp. in Europe and Transcaucasia are limited and often lack sufficient morphological descriptions or details on the fixation method (Manafov, 2011; Olenev, 1979; Zdun, 1961), making comparison difficult. Our study provides the first detailed morphometric analysis of genetically confirmed *M*. *romanicus* cercariae and demonstrates that fixation methods significantly alter biometric data. These results highlight the need for standardized fixation protocols, which is particularly important for future comparative studies, delimitation of species and interpretation of results in a taxonomic and ecological context.

In contrast to the narrow spectrum of snail hosts, *M. romanicus* can infect a very broad range of fish hosts in Europe, including no less than 52 fish species (Supplementary Table S5). Considering the extensive confusion with *M. yokogawai* (Scholz et al., 2024), regardless the differences in adult morphology, localization of metacercariae in fish (scales *vs* musculature) and geographical distribution, verified evidence of *M. romanicus* metacercariae in European fish is currently limited to the middle and lower section of the Danube from Slovakia to the Danube delta (including Bulgaria, Hungary, Romania, Serbia and Ukraine) (Fig. 1). In addition, this parasite is also well documented in other middle and lower river systems in Ukraine, especially in the Dnieper (Koval, 1950; Markevich, 1951), but also from the Southern Bug (Markevich, 1951) and the Dniester delta (Ciurea, 1933b; Zdun, 1961), which is consistent with the distribution area of suitable first snail intermediate hosts (Fig. 1).

Metacercariae of *M. romanicus* (misidentified as *M. yokogawai*) in chub have been reported from the Czech Republic in Brno at the confluence of the Svitava and Svratka, tributaries of the Morava River, which flows into the Danube about 100 km south of Bratislava (Lamková et al., 2007). However, the absence of voucher specimens and no detection of the parasite in fish in the nearby Morava and Dyje rivers in the present study suggest that this record should be interpreted with caution. Reliable evidence is also lacking for metacercariae reported from the European part of Russia, particularly from the middle and delta region of the Volga River (Molodozhnikova and Zhokhov, 2007), where introduction into fish farms near Moscow is suspected (Zhokhov et al., 2019), as the potential melanopsid snails have not been detected in the Volga basin (Starobogatov et al., 2004). Further records around the Caspian Sea (Semyonova et al., 2007), the Vyatka and Kama rivers (Grevtseva, 1979; Kostarev, 2003) and Dagestan (Engashev et al., 2021) also require re-evaluation due to the confirmed occurrence of *M. ciureanus* in the Transcaucasia region (Georgia) (see Scholz et al., 2024). All these reports suggest that *M. romanicus* is restricted to river systems in northern and western parts of the Black Sea basin, where suitable snail hosts are widely distributed (Fig. 1).

Within the broad spectrum of fish hosts, *M. romanicus* exhibits some preference for particular species from the family Leuciscidae, especially *Blicca* *bjoerkna* Heckel*, C. nasus, L. idus* and *S. cephalus* due to the highest infection intensity and prevalence (Cakić et al., 2007; Cech et al., 2023; Ciurea, 1915, Ciurea, 1933a; Koval, 1950; present study). Other species of this family (e.g., *Abramis brama* [L.] and *Alburnus alburnus* [L.]) can also harbour metacercariae with high prevalence, but the infection intensity is low (Cakić et al., 2007; Ciurea, 1933a; Koval, 1950; present study). Infections in fish species from other families (e.g., *Perca fluviatilis* L., *Neogobius melanostomus* [Pallas] and *Sander lucioperca* [L.]) are rare and of low intensity, suggesting that they are accidental hosts (Supplementary Tables S1 and S5) (Cakić et al., 2007; Cech et al., 2023; Ciurea, 1933a; Francová et al., 2011; Koval, 1950; present study).

In Europe, where raw fish is traditionally not consumed, no infections have been observed in humans. This is probably due to the exclusive localization of *M. romanicus* metacercariae in fish scales rather than in muscle tissue and the rare consumption of raw or undercooked fish. However, some risk remains and a small number of undiagnosed human infections cannot be ruled out due to accidental ingestion of scales during fish preparation and cleaning. In addition, pets such as dogs and cats can also become infected with metacercariae of *M. romanicus*. The experimental studies conducted by Rácz and Zemankovics (2002) and Gyöngy et al. (2024) on the effects of physical factors (such as freezing, heating and drying) and chemical factors (acetic acid and NaCl solution) commonly used in traditional food preservation methods on the viability of metacercariae of *M. romanicus* (misidentified as *M. yokogawai*) concluded that most of these treatments kill the metacercariae in a relatively short time, thus eliminating the risk of zoonotic infection. However, the metacercariae survived at room temperature and at 4 °C for up to one month. The storage of unprepared fish in household refrigerators therefore does not prevent infection with metacercariae in humans (Gyöngy et al., 2024).

## 5 Conclusion

We provide several new insights into the life cycle, distribution and transmission of *M*. *romanicus* and confirm *Microcolpia daudebartii acicularis* as the first intermediate host in Europe. We point out that certain *Melanopsis* species in Transcaucasia and the Middle East may serve as hosts for *M*. *ciureanus*, which may indicate a geographical separation of the two trematodes in the ecosystems of the European freshwater river systems of the Black Sea region. It is evident that the distribution of both species in their first hosts has been insufficiently studied so far, but that their potential for frequent occurrence in Europe is relatively high due to the large populations and wide distribution of the snail hosts. We also note that, in contrast to the limited range of the snail hosts, the metacercariae of *M. romanicus* occur in a wide variety of freshwater fish in Europe, with leuciscids being the most suitable hosts. To better understand the actual distribution of the two potentially zoonotic *Metagonimus* species, it is essential to use molecular methods to identify the cercariae in snails and the metacercariae in fish and to extend sampling in previously unexplored areas. Finally, a detailed morphological and morphometric description of *Metagonimus* cercariae, complemented by genetic data on both trematodes and snails, would facilitate future studies and improve our understanding of parasite circulation in European river catchments and tributaries.

## CRediT authorship contribution statement

**Mikuláš Oros:** Writing – original draft, Visualization, Investigation, Conceptualization. **Miroslava Soldánová:** Writing – original draft, Visualization, Methodology, Investigation, Conceptualization. **Daniel Barčák:** Writing – original draft, Methodology, Investigation. **Petra Kundid:** Writing – review & editing, Methodology, Investigation. **Caroline Jepkorir Kibet:** Methodology, Investigation. **Roman Kuchta:** Writing – original draft, Methodology, Investigation. **Martina Orosová:** Writing – review & editing, Methodology, Investigation. **Tomáš Scholz:** Writing – original draft, Visualization, Investigation, Conceptualization.

## Funding

This study was supported by the 10.13039/501100005357Slovak Research and Development Agency (APVV SK-CZ-RD-21-0078), Grant Agency of the Ministry of Education of the Slovak Republic and the Slovak Academy of Sciences (project No. VEGA 2/0130/24), the Ministry of Education, Youth and Sports of the Czech Republic (MEYS; project LUASK 22045), 10.13039/501100004240Czech Academy of Sciences (Mobility Plus Project SAV-23-08) and the Laboratory of Electron Microscopy, Institute of Parasitology, Biology Centre, CAS supported by the MEYS CR (LM2015062 Czech-Bioimaging).

## Declaration of competing interest

The authors declare that they have no known competing financial interests or personal relationships that could have appeared to influence the work reported in this paper.

## Appendix A Supplementary data

Supplementary material Image 1

## References

[bb0005] Abràmoff M.D., Magalhães P.J., Ram S.J. (2004). Image processing with Image. J. Biophotonics Int..

[bb0010] Arabuli L., Murvanidze L., Faltýnková A., Mumladze L. (2024). Checklist of digeneans (Platyhelminthes, Trematoda, Digenea) of Georgia. Biodivers. J..

[bb0015] Besprozvannykh V.V., Lebedev B.I. (1989). Investigation in Parasitology.

[bb0020] Besprozvannykh V.V. (2000).

[bb0025] Bikashvili A., Mumladze L., Glaubrecht M., Neiber M.T. (2025). Trapped in a glacial refugium: phylogeography of the freshwater snail *Melanopsis mingrelica* (Mollusca, Gastropoda) in the Caucasus biodiversity hotspot. Zool. Scr..

[bb0030] Bray R.A., Waeschenbach A., Cribb T., Weedall G., Dyal P., Littlewood D. (2009). The phylogeny of the Lepocreadioidea (Platyhelminthes, Digenea) inferred from nuclear and mitochondrial genes: implications for their systematics and evolution. Acta Parasitol..

[bb0035] Cakić P., Paunović M., Stojanović B., Ðikanović V., Kulišić Z. (2007). *Metagonimus yokogawai* (Katsurada, 1912), a new parasitic Trematoda species in ichtyoparasitofauna of the Serbia. Acta Vet. Brno.

[bb0040] Cech G., Gyöngy M., Sándor D., Kálmán M., Boglárka S., Ádám V., Csaba S. (2023). Molecular evidence of the absence of *Metagonimus yokogawai* (Katsurada, 1912) in Europe: report of *Metagonimus* sp. in cyprinoid fish from the river Danube in Hungary. Parasitol. Res..

[bb0045] Čejka, T., 2011. From the Red Book of our molluscs – *Esperiana*. Limno. Sprav. 5 1/2011. (in Czech).

[bb0050] Chai J.Y. (2019).

[bb0055] Chai J.Y., Jung B.K., Smithers G.W. (2024). Encyclopedia of Food Safety.

[bb0060] Chai J.Y., Darwin M., Lymbery A.J. (2005). Fish-borne parasitic zoonoses: status and issues. Int. J. Parasitol..

[bb0065] Chai J.Y., Shin E.H., Lee S.H., Rim H.J. (2009). Foodborne intestinal flukes in Southeast Asia. Korean J. Parasitol..

[bb0070] Chernomor O., von Haeseler A., Minh B.Q. (2016). Terrace aware data structure for phylogenomic inference from supermatrices. Syst. Biol..

[bb0075] Chervy L. (2024). Manual for the study of tapeworms (Cestoda) parasitic in ray-finned fish, amphibians and reptiles. Folia Parasitol..

[bb0080] Ciurea I. (1915). Über einige neue Distomen aus dem Darm unserer Haustiere und des Pelikans, für welche die Fische als Infektionsquelle zu betrachten sind. Zeit. Infekt. Parasit. Krank. Hyg. Haust..

[bb0085] Ciurea I. (1933). Les vers parasites de l’homme, des mammifères et des oiseaux provenant des poissons du Danube et de la mer Noire. Arch. Roum. Pathol. Exp. Microbiol..

[bb0090] Ciurea I. (1933). Sur quelques larves des Vers parasites de I’homme, des mammiferes et des Oiseaux ichtyopages, trouveés chez les poissons des grands lacs de la Bessarabie, du Dniester et de son Liman. Arch. Roum. Pathol. Exp. Microbiol..

[bb0095] Davis G.M. (1969). A taxonomic study of some species of *Semisulcospira* in Japan (Mesogastropoda: Pleuroceridae). Malacologia.

[bb0100] Dzhikia V.V. (1934).

[bb0105] Dzikowski R., Levy M.G., Poore M.F., Flowers J.R., Paperna I. (2004). Use of rDNA polymorphism for identification of Heterophyidae infecting freshwater fishes. Dis. Aquat. Org..

[bb0110] Engashev S.V., Shakhmurzov M.M., Bittirov A.M., Alieva K.G., Kaloshkina I.M., Medvedeva A.M. (2021). Epizootic and epidemic risks of the trematode *Metagonimus yokogawai* Katsurada, 1912 in Dagestan, as a biological threat to aquaculture of natural reservoirs and the population in the basin river Terek. Vet. Kubani.

[bb0115] Faltýnková A., Našincová V., Kablásková L. (2007). Larval trematodes (Digenea) of the great pond snail, *Lymnaea stagnalis* (L.), (Gastropoda, Pulmonata) in Central Europe: a survey of species and key to their identification. Parasite.

[bb0120] Faltýnková A., Našincová V., Kablásková L. (2008). Larval trematodes (Digenea) of planorbid snails (Gastropoda: Pulmonata) in Central Europe: a survey of species and key to their identification. Syst. Parasitol..

[bb0125] Faltýnková A., Sures B., Kostadinova A. (2016). Biodiversity of trematodes in their intermediate mollusc and fish hosts in the freshwater ecosystems of Europe. Syst. Parasitol..

[bb0130] Folmer O., Black M., Hoeh W., Lutz R., Vrijenhoek R. (1994). DNA primers for amplification of mitochondrial cytochrome *c* oxidase subunit I from diverse metazoan invertebrates. Mol. Mar. Biol. Biotechnol..

[bb0135] Francová K., Ondračková M., Polačik M., Jurajda P. (2011). Parasite fauna of native and non-native populations of *Neogobius melanostomus* (Pallas, 1814) (Gobiidae) in the longitudinal profile of the Danube River. J. Appl. Ichthyol..

[bb0140] Glöer P. (2019). Volume 1: Fresh- and Brakish Waters except Spring and Subterranean Snails.

[bb0145] Gradowski V.M. (1999). Trematodes of the molluscs of the family Melanopsidae (Gastropoda, Pectinibranchia, Cerithiiformes) from the Western Polesie area, with description of new cercaria. Vestn. Zool..

[bb0150] Grevtseva M.A. (1979).

[bb0155] Gyöngy M., Sellyei B., Czeglédi I., Székely C., Cech G. (2024). Viability of *Metagonimus romanicus* (Ciurea, 1915) metacercariae after physico-chemical treatments. Food Waterborne Parasitol..

[bb0160] Hoang D.T., Chernomor O., von Haeseler A., Minh B.Q., Vinh L.S. (2018). UFBoot2: improving the ultrafast bootstrap approximation. Mol. Biol. Evol..

[bb0165] Horsák M., Juřičková L., Picka J. (2013).

[bb0170] Iskova N.I., Sharpilo V.P., Sharpilo L.D., Tkach V.V. (1995).

[bb0175] Ismail N.S., Abdel-Hafez S.K., Toor M.A. (1983). Prevalence of gastrointestinal helminthes in cats from northern Jordan. Pak. Vet. J..

[bb0180] Ivasiuk Y., Losev A. (2019). Trematodes of gastropods of Kyiv reservoir. Sci. Issue Tern. Volod. H. Na.L Ped. Univ..

[bb0185] Kalyaanamoorthy S., Minh B.Q., Wong T.K.F., von Haeseler A., Jermiin L.S. (2017). ModelFinder: fast model selection for accurate phylogenetic estimates. Nat. Methods.

[bb0190] Kanarek G., Zaleśny G. (2014). Extrinsic- and intrinsic-dependent variation in component communities and patterns of aggregations in helminth parasites of great cormorant (*Phalacrocorax carbo*) from N.E. Poland. Parasitol. Res..

[bb0195] Kantor Y.I., Vinarski M.V., Schileyko A.A., Sysoev A.V. (2010). Catalogue of the Continental Mollusks of Russia and Adjacent Territories, Version 2.3.1. http://konstantinz.byethost32.com/books/kantor_2010.pdf?i=1.

[bb0200] Katoh K., Standley D.M. (2013). MAFFT multiple sequence alignment software version 7: improvements in performance and usability. Mol. Biol. Evol..

[bb0205] Kostarev G.F. (2003).

[bb0210] Koval B.P. (1950). Digenetic trematodes of fishes of the lower Dnieper. Tr. Biol. Fakul. Kyiv. Derzh. Univer..

[bb0215] Kozlov D.P. (1977).

[bb0220] Lamková K., Šimková A., Palíková M., Jurajda P., Lojek A. (2007). Seasonal changes of immunocompetence and parasitism in chub (*Leuciscus cephalus*), a freshwater cyprinid fish. Parasitol. Res..

[bb0225] Leonov V.A. (1960). Dynamics of the helminth fauna of the herring gull nesting on the territory of the Black Sea nature reserve. Uchen. Zap. Gorkov. Gosud. Pedag. Instit..

[bb0230] Littlewood D.T.J., Olson P.D., Littlewood D.T.J., Bray R.A. (2001). Interrelationships of the Platyhelminthes.

[bb0235] Ložek V. (1956).

[bb0240] Manafov A.A. (2011). Some results of the study of the trematode fauna of the freshwater mollusk *Melanopsis praemorsa* (L.) in the water bodies of Azerbaijan. Parazitologiya.

[bb0245] Markevich A.P. (1951).

[bb0250] Minh B.Q., Schmidt H.A., Chernomor O., Schrempf D., Woodhams M.D., von Haeseler A., Lanfear R. (2020). IQ-TREE 2: new models and efficient methods for phylogenetic inference in the genomic era. Mol. Biol. Evol..

[bb0255] Molodozhnikova N.M., Zhokhov A.E. (2007). The taxonomic diversity of the parasites of fishes in the Volga basin. III. Aspidogastrea and Trematoda. Parazitologiya.

[bb0265] Neiber M.T., Glaubrecht M. (2019). Unparalleled disjunction or unexpected relationships? Molecular phylogeny and biogeography of Melanopsidae (Caenogastropoda: Cerithioidea), with the description of a new family and a new genus from the ancient continent Zealandia. Cladistics.

[bb0270] Olenev A.V., Polyanski Y.I. (1979). Ecology and Experimental Parasitology.

[bb0275] Olson P.D., Cribb T.H., Tkach V.V., Bray R.A., Littlewood D.T.J. (2003). Phylogeny and classification of the Digenea (Platyhelminthes: Trematoda). Int. J. Parasitol..

[bb0280] Percie du Sert N., Hurst V., Ahluwalia A. (2020). The ARRIVE guidelines 2.0: updated guidelines for reporting animal research. BMC Vet. Res..

[bb0285] Rácz O.Z., Zemankovics E. (2002). Survival of metacercariae of *Metagonimus yokogawai* (Digenea: Heterophyidae) on fish from river Danube. Hung. Vet. J..

[bb0290] Rodonaya T.E. (1967).

[bb0295] Scholz T., Kuchta R., Barčák D., Cech G., Oros M. (2024). Small intestinal flukes of the genus *Metagonimus* (Digenea: Heterophyidae) in Europe and the Middle East: a review of parasites with zoonotic potential. Parasite.

[bb0300] Semyonova N.N., Ivanov V.P., Ivanov V.M. (2007).

[bb0305] Shumenko P.G., Tatonova Y.V., Besprozvannykh V.V. (2017). *Metagonimus suifunensis* sp. n. (Trematoda: Heterophyidae) from the Russian southern Far East: morphology, life cycle, and molecular data. Parasitol. Int..

[bb0310] Sitko J., Faltýnková A., Scholz T. (2006).

[bb0315] Soldánová M., Selbach C., Sures B., Kostadinova A., Pérez-Del-Olmo A. (2010). Larval trematode communities in *Radix auricularia* and *Lymnaea stagnalis* in a reservoir system of the Ruhr River. Parasit. Vectors.

[bb0320] Starobogatov Y.A.I., Prozorova L.A., Bogatov V.V., Saenko E.M., Tsalolikhin, S.Ya. (2004). Key to Freshwater Invertebrates of Russia and Adjacent Lands.

[bb0325] Strong E.E., Köhler F. (2009). Morphological and molecular analysis of ‘*Melania*’ *jacqueti* Dautzenberg and Fischer, 1906: from anonymous orphan to critical basal offshoot of the Semisulcospiridae (Gastropoda: Cerithioidea). Zool. Scr..

[bb0330] Tatonova Y.V., Shumenko P.G., Besprozvannykh V.V. (2013). Description of *Metagonimus pusillus* sp. nov. (Trematoda: Heterophyidae): phylogenetic relationships within the genus. J. Helminthol..

[bb0335] Tomović J., Paunović M., Atanacković A., Marković V., Gačić Z., Csányi B., Simić V. (2014). Biotic typology of the Danube River based on distribution of mollusc fauna as revealed by the second joint Danube survey (2007). Acta Zool. Bulgar..

[bb0340] Tytar V., Makarova N. (2015). Distribution of the freshwater snail species *Fagotia* (Gastropoda, Melanopsidae) in Ukraine according to climatic factors. I. *Fagotia esperi*. Vestn. Zool..

[bb0345] Vojtek J. (1974). Metacercariae from fishes of Czechoslovakia. Folia Fac. Sci. Nat. Univ. Purkynianae Brun..

[bb0350] Werle E., Schneider C., Renner M., Völker M., Fiehn W. (1994). Convenient single-step, one tube purification of PCR products for direct sequencing. Nucl. Acids Res..

[bb0355] Witenberg G. (1929). Studies on the trematode–family Heterophyidae. An. Trop. Med. Parasitol..

[bb0360] Xiao L., Ryan U., Feng Y. (2015).

[bb0365] Zdun V.I. (1961).

[bb0370] Zhokhov A.E., Pugacheva M.N., Molodozhnikova N.M., Berechikidze I.A. (2019). Alien parasite species of the fish in the Volga River basin: a review of data on the species number and distribution. Russ. J. Biol. Inv..

