# Supplementary material for: The life cycle of the potentially zoonotic trematode Metagonimus romanicus (Digenea: Heterophyidae): New insights from published and original data
Source: Food Waterborne Parasitol. 2025 Jul 5;40:e00276. doi: 10.1016/j.fawpar.2025.e00276 (PMC12281539; doi:10.1016/j.fawpar.2025.e00276)
Supplement: Supplementary file 1 — Supplementary material [file mmc1.docx]

**Supplementary Materials** (Supplementary Tables S1–S5 and Figs. S1–S3)

**The life cycle of the potentially zoonotic trematode *Metagonimus romanicus* (Digenea: Heterophyidae): New insights from published and original data**

Mikuláš Oros^a^, Miroslava Soldánová^b,*^, Daniel Barčák^a^, Petra Kundid^b,c^, Caroline Jepkorir Kibet^b,c^, Roman Kuchta^b^, Martina Orosová^a^, and Tomáš Scholz^b,c^

^a^ Institute of Parasitology, Slovak Academy of Sciences, Hlinkova 3, 040 01 Košice, Slovakia

^b^ Institute of Parasitology, Biology Centre, Czech Academy of Sciences, Branišovská 31, 370 05 České Budějovice, Czech Republic

^c^ Faculty of Science, University of South Bohemia in České Budějovice, Branišovská 31a, 370 05 České Budějovice, Czech Republic

^*^ Corresponding author.

*E-mail address:* [soldanova@paru.cas.cz](mailto:soldanova@paru.cas.cz) (M. Soldánová), Institute of Parasitology, Biology Centre, Czech Academy of Sciences, Branišovská 31, 370 05 České Budějovice, Czech Republic, +420 387775484

**Supplementary Table S1.** Summary of fish examined for metacercariae of *Metagonimus romanicus* (Ciurea, 1915) in scales of fish. Data are presented as number of fish individuals examined/number of positive fish individuals.

| **Fish host/country** | **Austria** | **Czech Republic** | **Hungary** | **Slovakia** | **All countries** |
| --- | --- | --- | --- | --- | --- |
| **Cyprinidae** |  |  |  |  |  |
| *Barbus barbus* | 9/0 | 5/0 | 1/0 | 1/0 | 16/0 |
| *Carassius carassius* | – | 5/0 | – | – | 5/0 |
| *Carassius gibelio* | – | – | – | 4/0 | 4/0 |
| *Cyprinus carpio* | – | – | – | 1/0 | 1/0 |
| **Esocidae** |  |  |  |  |  |
| *Esox lucius* | – | – | – | 1/0 | 1/0 |
| **Gobiidae** |  |  |  |  |  |
| *Babka gymnotrachelus* | – | – | – | 2/0 | 2/0 |
| *Neogobius melanostomus* | – | – | 1/0 | 1/1 | 2/1 |
| *Ponticola kessleri* | – | – | – | 2/0 | 2/0 |
| **Gobionidae** |  |  |  |  |  |
| *Gobio gobio* | – | 1/0 | 1/0 | – | 2/0 |
| *Pseudorasbora parva* | – | – | – | 1/0 | 1/0 |
| **Leuciscidae** |  |  |  |  |  |
| *Abramis brama* | – | 5/0 | – | 2/1 | 7/1 |
| *Alburnus alburnus* | 2/0 | 9/0 | 1/1 | 7/7 | 19/8 |
| *Ballerus sapa* | – | – | – | 2/2 | 2/2 |
| *Blicca bjoerkna* | – | – | – | 2/1 | 2/1 |
| *Chondrostoma nasus* | – | 1/0 | 5/5 | 11/10 | 17/15 |
| *Leuciscus aspius* | – | – | – | 2/1 | 2/1 |
| *Leuciscus idus* | – | – | – | 7/6 | 7/6 |
| *Rhodeus amarus* | – | – | – | 5/0 | 5/0 |
| *Rutilus rutilus* | – | 1/0 | – | 5/5 | 6/5 |
| *Scardinius erythrophthalmus* | – | 6/0 | – | 5/1 | 11/1 |
| *Squalius cephalus* | 3/0 | 26/0 | 19/17 | 7/6 | 55/23 |
| *Vimba vimba* | – | – | 1/0 | – | 1/0 |
| **Percidae** |  |  |  |  |  |
| *Gymnocephalus cernua* | – | – | – | 1/0 | 1/0 |
| *Perca fluviatilis* | – | – | 1/0 | 5/1 | 6/1 |
| *Sander lucioperca* | – | – | – | 1/0 | 1/0 |
| **Tincidae** |  |  |  |  |  |
| *Tinca tinca* | – | – | – | 1/0 | 1/0 |
| **Total** | **14/0** | **59/0** | **30/23** | **76/42** | **179/65** |

**Supplementary Table S2.** Summary of sequences used in phylogenetic analysis with newly generated sequences in bold.

| **Species (isolate)** | **Stage** | **Experimental host** | **Natural host** | **Country** | **18S rDNA** | **28S rDNA** | **ITS** | **Reference** |
| --- | --- | --- | --- | --- | --- | --- | --- | --- |
| *Metagonimus ciureanus** | A | N/A | *Phalacrocorax carbo* | Israel | AY245702 | N/A | AY245702 | Dzikowski et al., 2004 |
| *M. hakubaensis* | A | *Mesocricetus auratus* | *Lethenteron reissneri* | Japan | N/A | KM061389 | KM061398 | Pornruseetairatn et al., 2016 |
| *M. katsuradai* | A | *Mesocricetus auratus* | *Tanakia limbata* | Japan | N/A | KM061391 | KM061400 | Pornruseetairatn et al., 2016 |
| *M. kinoi* | R | N/A | *Semisulcospira libertina* | Japan | N/A | LC599533 | N/A | Nakao et al., 2022 |
| *M. miyatai* | A | *Mesocricetus auratus* | *Plecoglossus altivelis* | Japan | HQ832624 | HQ832633 | HQ832615 | Pornruseetairatn et al., 2016 |
| *M. otsurui* | A | *Mesocricetus auratus* | *Rhinogobius flumineus* | Japan | N/A | KM061394 | KM061404 | Pornruseetairatn et al., 2016 |
| *M. pusillus* | A | *Anas platyrhynchos* | *Rhodeus sericeus* | Russia | N/A | MF407172 | MF407172 | Tatonova et al., 2013 |
| *M. romanicus* HU24-2 | A | *Mesocricetus auratus* | *Squalius cephalus* | Hungary | PP378514 | PP378508 | N/A | Scholz et al., 2024 |
| *M. romanicus* MM2**** | M | N/A | *Scardinius erythrophthalmus* | Hungary | N/A | OQ286071 | OQ286093 | Cech et al., 2023 |
| *M. romanicus* SPM2**** | M | N/A | *Perca fluviatilis* | Hungary | N/A | OQ286076 | N/A | Cech et al., 2023 |
| ***M. romanicus*** SK45-1 | M | N/A | *Leuciscus aspius* | Slovakia | N/A | **PV777974** | **PV779653** | **Present study** |
| ***M. romanicus*** SK841-2 | M | N/A | *Squalius cephalus* | Slovakia | N/A | **PV777975** | **PV779654** | **Present study** |
| ***M. romanicus*** SK843-1 | M | N/A | *Leuciscus idus* | Slovakia | N/A | **PV777976** | **PV779655** | **Present study** |
| ***M. romanicus*** SK846-2 | M | N/A | *Alburnus alburnus* | Slovakia | N/A | **PV777977** | **PV779656** | **Present study** |
| ***M. romanicus*** SK855-3 | M | N/A | *Blicca bjoerkna* | Slovakia | N/A | **PV777978** | **PV779657** | **Present study** |
| ***M. romanicus*** SK860-1 | M | N/A | *Chondrostoma nasus* | Slovakia | N/A | **PV777979** | **PV779658** | **Present study** |
| ***M. romanicus*** SK863-2 | M | N/A | *Rutilus rutilus* | Slovakia | N/A | **PV777980** | **PV779659** | **Present study** |
| ***M. romanicus*** SK894-6 | M | N/A | *Neogobius melanostomus* | Slovakia | N/A | **PV777981** | **PV779660** | **Present study** |
| ***M. romanicus*** SK902-1 | M | N/A | *Ballerus sapa* | Slovakia | N/A | **PV777982** | **PV779661** | **Present study** |
| ***M. romanicus*** MS7 | C | N/A | *Microcolpia daudebartii acicularis* | Slovakia | N/A | **PV777983** | **PV779662** | **Present study** |
| ***M. romanicus*** M4-1 | C | N/A | *Microcolpia daudebartii acicularis* | Slovakia | N/A | N/A | **PV779663** | **Present study** |
| *M. saitoi* | M | N/A | *Opsariichthys platypus* | Japan | N/A | LC666746 | N/A | Nakao et al., 2022 |
| *M. shimazui* | M | N/A | *Acheilognathus rhombeus* | Japan | N/A | LC66675 | N/A | Nakao et al., 2022 |
| *M. suifunensis* | A | *Rattus norvegicus* | *Parajuga subextensa* | Russia | N/A | KX387459 | KX387484 | Shumenko et al., 2017 |
| *M. takahashii* | A | *Mesocricetus auratus* | *Carassius langsdorfii* | Japan | HQ832627 | HQ832636 | HQ832618 | Pornruseetairatn et al., 2016 |
| *M. yokogawai* | A | *Mesocricetus auratus* | *Plecoglossus altivelis* | Japan | HQ832630 | HQ832639 | HQ832621 | Pornruseetairatn et al., 2016 |
| **Outgroup** |  |  |  |  |  |  |  |  |
| *Cryptocotyle lingua* | C | N/A | *Littorina littorea* | Germany | AJ287492 | AY222228 | N/A | Littlewood et al., 2001 |
| *Euryhelmis costaricensis* | M | N/A | *Hynobius lichenatus* | Japan | AB521797 | AB521797 | AB521797 | Sato et al., 2010 |
| *Metagonimoides oregonensis* | C | N/A | *Pleurocera proxima* | USA | N/A | JQ995473 | N/A | Belden et al., 2012 |

* Reported as *Dexiogonimus ciureanus* and ** as *Metagonimus* sp. in GenBank. *Abbreviations*: A, adult; C, cercaria; M, metacercaria; R, redia;

N/A, not available.

**Supplementary Table S3.** Comparison of published dimensions of cercariae of *Metagonimus* spp. Data (in micrometres) are presented as mean followed by a range in parentheses.

| **Species** | ***Metagonimus* ‘*yokogawai*’** | ***Metagonimus* ‘*yokogawai*’** | ***Cercaria metagonimus* sp.** | ***Cercaria metagonimus* ‘*yokogawai*’** |
| --- | --- | --- | --- | --- |
| **Snail host** | *Microcolpia daudebartii acicularis*,  *Esperiana esperi** | *Melanopsis praemorsa* | *Melanopsis* *praemorsa* | *Melanopsis praemorsa* |
| **Locality/country** | Dniester River/Ukraine | Supsa, Natanebi & St. Pichora rivers/ Georgia | Kura River/  Azerbaijan | Kura River/  Azerbaijan |
| **Reference** | Zdun, 1961 | Olenev, 1979 | Manafov, 2011 | Manafov, 2011 |
| **Fixation method** | N/A | hot formalin | hot formalin | hot formalin |
| ***N* cercariae** | N/A | N/A | 15 | 15 |
| Total length | 500** | (470–580)** | 626 (523–652)** | (455–554)** |
| Body length (BL) | 200 | (150–180) | 183 (146–189) | (158–180) |
| Body width (BW) | 50 | (70–80) | 91 (88–94) | (80–87) |
| Tail length (TL) | 300 | (320–400) | 443 (377–463) | (297–374) |
| Tail width (TW) | 30 | (20–30) | (38–44) | (55–66) |
| Oral sucker length (OSL) | – | 40 | – | 47 |
| Oral sucker width (OSW) | 40 | 40 | 38 (36–42) | 39 |
| Pharynx length (PL) | – | – | – | – |
| Pharynx width (PW) | – | – | 14 (12–18) | – |
| Ventral sucker length (VSL) | – | – | – | – |
| Ventral sucker width (VSW) | – | – | 24 (21–27) | – |
| Tail length/body length (ratio) | 1.5** | (2.1–2.2)** | 2.4 (2.5–2.6)** | (1.8–2.1)** |
| Oral/ventral sucker width (ratio) | – | – | 1.6 (1.6–1.7)** | – |

* Reported as *Fagotia acicularis*, *F. esperi*. *Abbreviations*: N/A, not available. ** Morphometric parameters calculated from the values in the original article. See Supplementary Fig. S1 for an illustration and abbreviations of the morphometric features.

**Cercariae: Supplementary remarks to section 3.3.** (Description of cercariae)

Only a few previous records have identified cercariae of *Metagonimus* spp. from melanopsid snails in Europe and Transcaucasia based solely on morphology. These include cercariae from *M. daudebartii acicularis* (identified as *Fagotia acicularis*) and *Esperiana esperi* (Férussac) (as *F. esperi*) in Ukraine (Zdun, 1961) and cercariae from *Melanopsis praemorsa* (probably *M*. *mingrelica* Mousson, whose occurrence in both countries was confirmed by molecular data (Bikashvili et al., 2025)) in Georgia (Olenev, 1979) and Azerbaijan (Manafov, 2011) (Supplementary Table S3).

The present cercariae closely match those described in previous studies, as they exhibit features of pleurolophocercous cercariae, including a long, simple tail, a pair of pigmented eye-spots, a prominent dorsoventral undulating fin-fold, a spinous body, a subspherical oral sucker with oral spines, seven pairs of penetration gland-cells, and a Y-shaped excretory bladder. The total body length (sum of body and tail length) of our *M*. *romanicus* cercariae is within the range reported in studies using hot formalin (Table 1; Supplementary Table S3).

Although largely consistent, our specimens show some biometric and morphological differences. The cercariae of *M*. ‘*yokogawai*’ from Zdun (1961) are generally smaller (about 60 μm), especially in body width and tail length, although they share features such as pigmented eye-spots, number of penetration glands, hook-like oral spines and dorsoventral fin-fold. The present cercariae are most similar to *M*. ‘*yokogawai*’ of Olenev (1979), differing slightly in the shape of oral sucker and tail length (about 40 μm shorter). Both Olenev’s and our specimens show a similar arragement of oral spines in three rows (four larger ones in the first row and 11–14 smaller ones in the following rows), but differ in the tegumental spination (reaching the posterior end of the oral sucker *vs* spines covering the entire body in our cercariae). *Cercaria metagonimus* *yokogawai* Takahashi, 1929 (referred to as *M*. ‘*yokogawai*’ for simplicity) by Manafov (2011) also overlap metrically, but are generally smaller and have a much shorter (about 40–95 μm) and broader tail (difference of ca. 30 μm).

The most striking differences are with *Cercaria metagonimus* sp. (referred to as *Metagonimus* sp. for simplicity) from Manafov (2011), which are larger in all metric parameters (Table 1; Supplementary Table S3), have a different arrangement of oral spines (in 4 rows, consisting of 4 : 8 : 12 : 14 spines), but share full-body tegmental spination.

**Rediae: Supplementary remarks to section 3.4.** (Description of rediae)

The rediae of *M*. ‘*yokogawai*’ described by Zdun (1961) are shorter and much broader (average size 800 × 200 μm), but no details of their morphology and fixation method were provided. Mature daughter rediae of *M*. ‘*yokogawai*’ described by Olenev (1979) are much smaller (500–600 × 100–200 μm) than the present rediae, although they overlap in width but lack the constriction in the pharyngeal region. The daughter rediae of *Metagonimus* sp. from Manafov (2011) overlap in dimensions (440–935 × 110–143 μm) with the present rediae, although their pharynx is smaller (diameter 29–33 μm) compared to the rediae in this study.

**Supplementary Table S4.** Results of separate one-way ANOVA tests assessing the differences in morphometric parameters of cercariae of *Metagonimus romanicus* (Ciurea, 1915) among three different methods (live cercariae, cercariae fixed in hot and cold formalin), followed by pairwise comparison using Tukey’s HSD *post-hoc* tests. Statistically significant results (*P* < 0.05) are in bold.

| **Parameter tested** | **df** | **MS** | **F** | ***P*** | ***Post-hoc* test** | ***P*** |
| --- | --- | --- | --- | --- | --- | --- |
| Body length (BL) | 2 | 0.853 | 67.7 | **< 0.01** | L > H > C | **all < 0.001** |
| Body width (BW) | 2 | 1.318 | 111.9 | **< 0.01** | L > H, L > C, H < C | **all < 0.001** |
| Tail length (TL) | 2 | 0.096 | 13.4 | **< 0.001** | L > H, L = C, H < C | **< 0.001**, > 0.05, **< 0.01** |
| Tail width (TW) | 2 | 0.009 | 0.6 | 0.57 | L = H = C | > 0.05 |
| Tail socket length (TSL) | 2 | 1.358 | 61.3 | **< 0.01** | L > H, L > C, H = C | **< 0.001**, **< 0.001**, > 0.05 |
| Oral sucker length (OSL) | 2 | 0.547 | 83.2 | **< 0.01** | L > H, L > C, H = C | **< 0.001**, **< 0.001**, > 0.05 |
| Oral sucker width (OSW) | 2 | 0.461 | 33.8 | **< 0.001** | L > H, L > C, H = C | **< 0.001**, **< 0.001**, > 0.05 |
| Eye-spots to anterior body end (ESbe) | 2 | 4.586 | 166.9 | **< 0.01** | L > H > C | **all < 0.001** |
| Eye-spots diameter (ESd) | 2 | 0.329 | 23.11 | **< 0.001** | L > H, L > C, H < C | **< 0.001**, **< 0.001**, **< 0.05** |

*Abbreviations*: df, degrees of freedom; MS, means of squares; F, test criterion value; *P*, probability value; L, live cercariae; H, cercariae fixed in hot formalin; C, cercariae fixed in cold formalin. See Supplementary Figure S1 for an illustration and abbreviations of the morphometric features.

**Metacercariae: Supplementary remarks to section 3.7.** (Description of metacercariae)

The present study confirms that metacercariae of *M. romanicus* are widespread in fish along the middle part of the Danube, with a higher prevalence of infection in Slovakia than previously reported in the Hungarian Danube by Cech et al. (2023). However, no less than 52 freshwater fish species from 18 families (Supplementary Table S5) have been reported as hosts for *M. romanicus* (identified as *M.* ‘*yokogawai*’) in Bulgaria (Francová et al., 2011; Kakacheva-Avramova, 1983), the Czech Republic (Lamková et al., 2007), Hungary (Cech et al., 2023), Romania (Ciurea, 1933), Serbia (Cakić et al., 2007), Slovakia (Moravec, 2001) and Ukraine (Bychovskaya-Pavlovskaya, 1962 – Dnieper; Ciurea, 1933; Koval, 1950; Kulakovskaya, 1955 – Seret River; Kulakovskaya and Koval, 1973 – Danube delta; Malevitskaya, 1938; Markevich, 1951 – Southern Bug River) (Fig. 1).

The dimensions of our live cysts correspond to previous records from Romania (Ciurea, 1933), Slovakia (Vojtek, 1974) and Ukraine (Markevich, 1951), while the ethanol-fixed cysts described in the present study were slightly smaller than the live cysts, probably due to shrinkage of the parasite and distortion of the cyst shape. The high intensity of cysts in some scales of nase, chub and ide (Table 2) has also been reported previously in leuciscids in Ukraine (e.g., 99 cysts in a chub scale (Koval, 1950), 48 in bream and 37 in rudd (Malevitskaya, 1938)).

The excysted metacercariae from the scales of *Blicca* *bjoerkna* Heckel, nase and chub correspond morphologically well with the metacercariae of eight fish species from the type locality in Romania (Ciurea, 1933) and the middle Danube in Slovakia (Vojtek, 1974). The distribution of the spines over the entire body surface (with the exception of the areas of the oral and ventral suckers), the relative distance of the ventral sucker from the anterior extremity and the diagonal position of the testes correspond to species-specific morphological characteristics of the adults (Scholz et al., 2024).

Compared to *M.* ‘*yokogawai*’, the excysted metacercariae of *M. romanicus* appear similar, but differ slightly in the morphology of their anterior body parts. *Metagonimus romanicus* has a more slender anterior half of the body (83–115 µm wide *vs* 163–190 µm in *M.* ‘*yokogawai*’), a larger oral sucker (41–52 × 43–55 µm *vs* 32–35 × 34–38 µm) and a longer oesophagus (36–53 µm *vs* 29–32 µm (see Hong and Seo, 1969). These features generally correspond to differences seen in adults, including the greater distance of the ventrogenital sac from the anterior tip of the body in *M. romanicus* compared to *M.* ‘*yokogawai*’ (Scholz et al., 2024).

**Supplementary Table S5**. Summary of fish species reported as second intermediate hosts of *Metagonimus romanicus* (Ciurea, 1915) (often misidentified as *M*. ‘*yokogawai*’) in Europe.

| **No.** | **Fish species** | **Fish family** | **Reference** |
| --- | --- | --- | --- |
| 1. | *Abramis brama* | Leuciscidae | Moravec, 2001 |
| 2. | *Acipenser ruthenus* | Acipenseridae | Moravec, 2001 |
| 3. | *Alburnoides bipunctatus* | Leuciscidae | Moravec, 2001 |
| 4. | *Alburnus alburnus* | Leuciscidae | Moravec, 2001 |
| 5. | *Ameiurus nebulosus* | Ictaluridae | Moravec, 2001 |
| 6. | *Anguilla anguilla* | Anguillidae | Moravec, 2001 |
| 7. | *Ballerus* *ballerus* | Leuciscidae | Moravec, 2001 |
| 8. | *Ballerus sapa* | Leuciscidae | Moravec, 2001 |
| 9. | *Barbatula barbatula* | Nemacheilidae | Moravec, 2001 |
| 10. | *Barbus barbus* | Cyprinidae | Moravec, 2001 |
| 11. | *Barbus petenyi* | Cyprinidae | Moravec, 2001 |
| 12. | *Blicca bjoerkna* | Leuciscidae | Moravec, 2001 |
| 13. | *Carassius auratus* | Cyprinidae | Moravec, 2001 |
| 14. | *Carassius carassius* | Cyprinidae | Moravec, 2001 |
| 15. | *Carassius gibelio* | Cyprinidae | Morozov, 1952 |
| 16. | *Chondrostoma nasus* | Leuciscidae | Moravec, 2001 |
| 17. | *Cobitis taenia* | Cobitidae | Moravec, 2001 |
| 18. | *Ctenopharyngodon idella* | Xenocyprididae | Moravec, 2001 |
| 19. | *Cyprinus carpio* | Cyprinidae | Moravec, 2001 |
| 20. | *Esox lucius* | Esocidae | Moravec, 2001 |
| 21. | *Gobio gobio* | Gobionidae | Moravec, 2001 |
| 22. | *Gymnocephalus cernua* | Percidae | Moravec, 2001 |
| 23. | *Gymnocephalus schraetser* | Percidae | Moravec, 2001 |
| 24. | *Hypophthalmichthys molitrix* | Xenocyprididae | Morozov, 1952 |
| 25. | *Lepomis gibbosus* | Centrarchidae | Moravec, 2001 |
| 26. | *Leucaspius delineates* | Leuciscidae | Moravec, 2001 |
| 27. | *Leuciscus aspius* | Leuciscidae | Moravec, 2001 |
| 28. | *Leuciscus cephalus* | Leuciscidae | Moravec, 2001 |
| 29. | *Leuciscus idus* | Leuciscidae | Moravec, 2001 |
| 30. | *Leuciscus leuciscus* | Leuciscidae | Moravec, 2001 |
| 31. | *Lota lota* | Lotidae | Moravec, 2001 |
| 32. | *Misgurnus fossilis* | Cobitidae | Moravec, 2001 |
| 33. | *Neogobius melanostomus* | Gobiidae | Francová et al., 2011 |
| 34. | *Pelecus cultratus* | Leuciscidae | Moravec, 2001 |
| 35. | *Perca fluviatilis* | Percidae | Moravec, 2001 |
| 36. | *Percarina demidoffii* | Percidae | Ciurea, 1933 |
| 37. | *Petroleuciscus borysthenicus* | Leuciscidae | Morozov, 1952 |
| 38. | *Phoxinus phoxinus* | Leuciscidae | Moravec, 2001 |
| 39. | *Rhodeus sericeus* | Acheilognathidae | Moravec, 2001 |
| 40. | *Romanogobio albipinnatus* | Gobionidae | Moravec, 2001 |
| 41. | *Romanogobio kesslerii* | Gobionidae | Moravec, 2001 |
| 42. | *Romanogobio uranoscopus* | Gobionidae | Moravec, 2001 |
| 43. | *Rutilus pigus* | Leuciscidae | Moravec, 2001 |
| 44. | *Rutilus rutilus* | Leuciscidae | Moravec, 2001 |
| 45. | *Sander* *lucioperca* | Percidae | Moravec, 2001 |
| 46. | *Sander* *volgense* | Percidae | Moravec, 2001 |
| 47. | *Scardinius erythrophthalmus* | Leuciscidae | Moravec, 2001 |
| 48. | *Silurus glanis* | Siluridae | Moravec, 2001 |
| 49. | *Syngnathus abaster* | Syngnathidae | Ondračková et al., 2012 |
| 50. | *Tinca tinca* | Tincidae | Moravec, 2001 |
| 51. | *Vimba vimba* | Leuciscidae | Moravec, 2001 |
| 52. | *Zingel zingel* | Percidae | Moravec, 2001 |


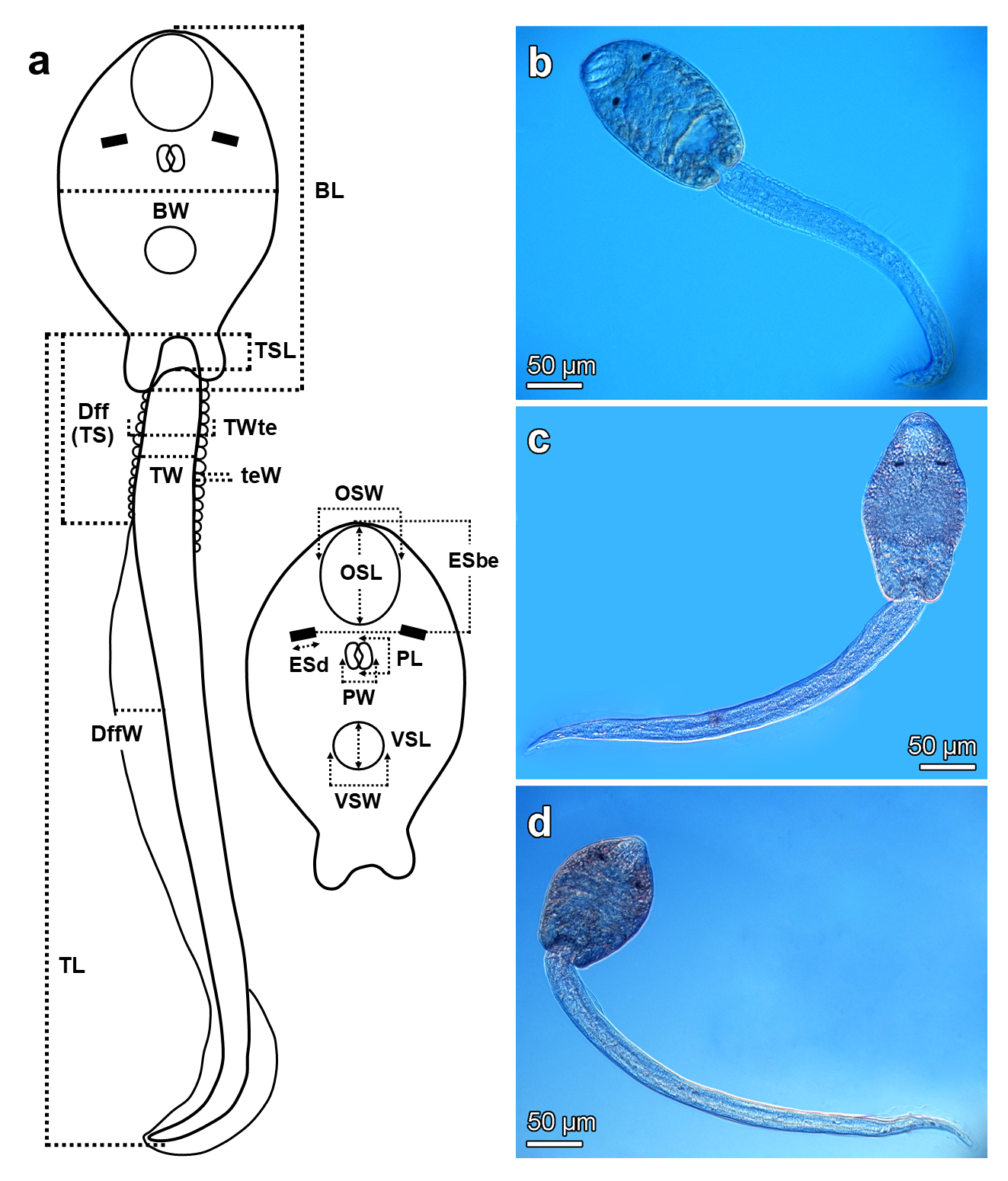


**Supplementary Fig. S1. (a)** Schematic illustration of a cercaria of *Metagonimus romanicus* (Ciurea, 1915) showing the morphometric features used. *Abbreviations*: BL, body length; BW, body width; Dff(TS), dorsal fin-fold – start from the tail socket; DffW, dorsal fin-fold width; ESbe, eye-spots to anterior body end; ESd, eye-spots diameter; OSL, oral sucker length; OSW, oral sucker width; PL, pharynx length; PW, pharynx width; teW, tegument width; TL, tail length; TW, tail without tegument width; TWte, tail with tegument width; TSL; tail socket length; VSL, ventral sucker length; VSW, ventral sucker width. Total view of **(b)** live cercaria **(c)** cercaria fixed in hot formalin **(d)** cercaria fixed in cold formalin.


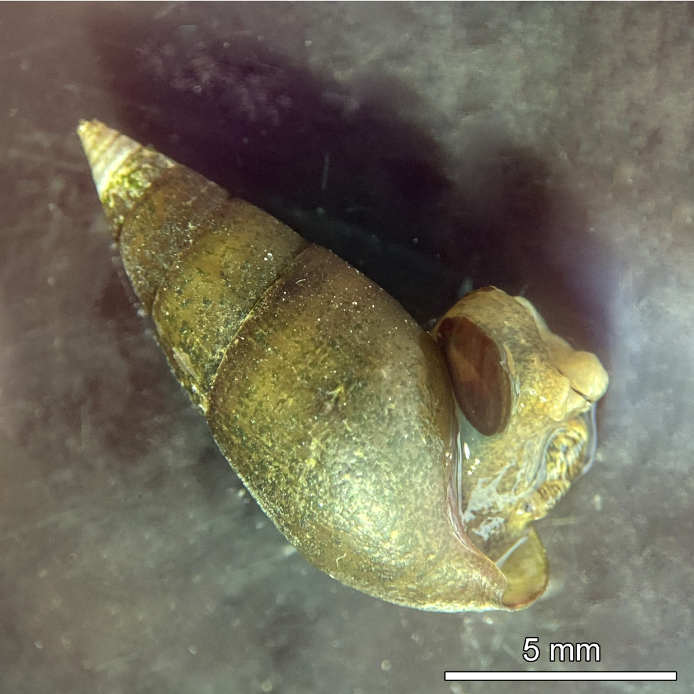


**Supplementary Fig. S2.** *Microcolpia daudebartii acicularis* (Férussac), the snail host of the trematode *Metagonimus romanicus* (Ciurea, 1915), Danube, Slovakia (Bočianské arm near the town Gabčíkovo).


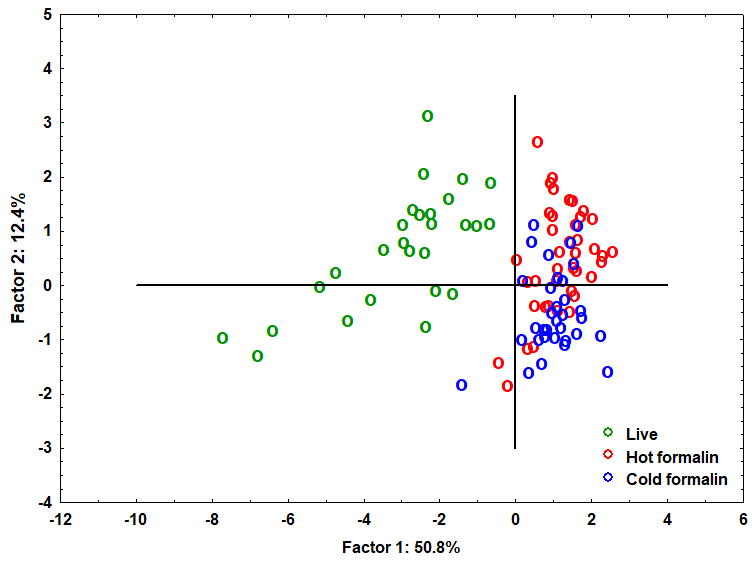


**Supplementary Fig. S3.** Principal component analysis (PCA) scores plot showing the distribution of individual cercariae of *Metagonimus romanicus* (Ciurea, 1915), isolated from the snail host *Microcolpia daudebartii acicularis* (Férussac), based on nine morphometric features across three fixation methods along the first and second principal component (PC1 plotted on the x-axis and PC2 plotted on the y-axis).

**References**

Belden, K.L., Peterman, W.E., Smith, S.A., Brooks, L.R., Benfield, E.F., Black, W.P., Yang, Z., Wojdak, J.M., 2012. *Metagonimoides oregonensis* (Heterophyidae: Digenea) infection in Pleurocerid snails and *Desmognathus quadramaculatus* salamander larvae in Southern Appalachian streams*.* J. Parasitol. 98,760–767. <https://doi.org/10.1645/GE-2986.1>.

Bikashvili, A., Mumladze, L., Glaubrecht, M., Neiber, M.T., 2025. Trapped in a glacial refugium: Phylogeography of the freshwater snail *Melanopsis mingrelica* (Mollusca, Gastropoda) in the Caucasus biodiversity hotspot. Zool. Scr. 54,332–351. <https://doi.org/10.1111/zsc.12713>.

Bychovskaya-Pavlovskaya, I.E., 1962. Trematodes of the birds of the fauna of the USSR. Akademia Nauk SSSR, Moscow. (in Russian)

Cakić, P., Paunović, M., Stojanović, B., Ðikanović, V., Kulišić, Z., 2007. *Metagonimus yokogawai* (Katsurada, 1912), a new parasitic Trematoda species in ichtyoparasitofauna of the Serbia. Acta Vet. 57,537–543. <https://doi.org/10.2298/avb0706537c>.

Cech, G., Gyöngy, M., Sándor, D., Kálmán, M., Boglárka, S., Ádám, V., Csaba, S., 2023. Molecular evidence of the absence of *Metagonimus yokogawai* (Katsurada, 1912) in Europe: report of *Metagonimus* sp. in cyprinoid fish from the River Danube in Hungary. Parasitol. Res. 122,2325–2334. <https://doi.org/10.1007/s00436-023-07932-1>.

Ciurea, I., 1933. Les vers parasites de l’homme, des mammifères et des oiseaux provenant des poissons du Danube et de la mer Noire. Arch. Roum. Pathol. Exp. Microbiol. 6,5–134.

Dzikowski, R., Levy, M.G., Poore, M.F., Flowers, J.R., Paperna, I., 2004. Use of rDNA polymorphism for identification of Heterophyidae infecting freshwater fishes. Dis. Aquat. Organ. 59,35–41. <https://doi.org/10.3354/dao059035>.

Francová, K., Ondračková, M., Polačik, M., Jurajda, P., 2011. Parasite fauna of native and non-native populations of *Neogobius melanostomus* (Pallas, 1814) (Gobiidae) in the longitudinal profile of the Danube River. J. Appl. Ichthyol. 27,879–886. <https://doi.org/10.1111/j.1439-0426.2010.01582.x>.

Hong, N.T., Seo, B.S. 1969. Study of *Metagonimus yokogawai* (Katsurada, 1912) in Korea I. On the metacercaria, its distribution in the second intermediate host and the development in the final host. Kor. J. Parasitol. 7,129–142. <http://doi.org/10.3347/kjp.1969.7.3.129>.

Kakacheva-Avramova D., 1983. Helminths of Freshwater Fishes in Bulgaria, Bulgarian Academy of Science, Sofia. (in Russian)

Koval, B.P., 1950. Digenetic trematodes of fishes of the lower Dnieper. Tr. Biol. Fakul. Kyiv. Derzh. Univer. 5,187–207. (in Russian)

Kulakovskaya, O.P., 1955. Parasites of fishes of the upper Dniester basin. Dissertation, Academia Nauk USSR, Lviv. (in Russian)

Kulakovskaya, O.P., Koval, V.P., 1973. Parasitofauna of fishes from Danube River basin. Naukova Dumka, Kyiv. (in Russian)

Lamková, K., Šimková, A., Palíková, M., Jurajda, P., Lojek, A., 2007. Seasonal changes of immunocompetence and parasitism in chub (*Leuciscus cephalus*), a freshwater cyprinid fish. Parasitol. Res. 101,775–789. <https://doi.org/10.1007/s00436-007-0546-3>.

Littlewood, D.T.J., Olson, P.D., 2001. Small subunit rDNA and the Platyhelminthes: Signal, noise, conflict and compromise, in: Littlewood, D.T.J., Bray, R.A. (Eds.), Interrelationships of the Platyhelminthes. Taylor & Francis, London, pp. 262–278. <https://doi.org/10.1201/9781482268218>.

Malevitskaya, M.A., 1938. On the question of the presence of *Metagonimus yokogawai* Katsurada on the territory of the Ukrainian SSR. Zbir. Prats Zool. Muz. Ukr. Akad. Nauk 21–22,87–192. (in Russian)

Manafov, A.A., 2011. Some results of the study of the trematode fauna of the freshwater mollusk *Melanopsis praemorsa* (L.) in the water bodies of Azerbaijan. Parazitologiya 45,367–378. (in Russian)

Markevich, A.P., 1951. Parasite Fauna of Freshwater Fish of the Ukrainian SSR. Izdavatelstvo Akademii Nauk USSR, Kyiv. (in Russian)

Moravec F., 2001. Checklist of the Metazoan Parasites of Fishes of the Czech Republic and the Slovak Republic (1873–2000). Academia, Prague.

Morozov, F.N., 1952. Trematode superfamily Heterophyoidea Faust, 1929, in: Skryabin, K.I. (Ed.), Osnovy Trematodologii (Trematody Zhivotnykh i Cheloveka) Vol. 6. Akademia Nauk SSSR, Moscow, pp. 152–615. (in Russian)

Nakao, M., Ishikawa, T., Hibino, Y., Ohari, Y., Taniguchi, R., Takeyama, T., Nakamura, S., Kakino, W., Ikadai, H., Sasaki, M., 2022. Resolution of cryptic species complexes within the genus *Metagonimus* (Trematoda: Heterophyidae) in Japan, with description of four new species. Parasitol. Int. 90,1–14. <https://doi.org/10.1016/j.parint.2022.102605>.

Olenev, A.V., 1979. Cercarial fauna of the freshwater mollusk *Melanopsis praemorsa* (L.) from Western Georgia., in: Polyanski, Y.I. (Ed.), Ecology and Experimental Parasitology. Leningradskiy Gosudarstvennii Universitet, St. Petersburg, pp. 30–41. (in Russian)

Ondračková, M., Slováčková, I., Trichkova, T., Polačik, M., Jurajda, P., 2012. Shoreline distribution and parasite infection of black-striped pipefish *Syngnathus abaster* Risso, 1827 in the lower River Danube. J. Appl. Ichthyol. 28,590–596. <https://doi.org/10.1111/j.1439-0426.2012.01967.x>.

Pornruseetairatn, S., Kino, H., Shimazu, T., Nawa, Y., Scholz, T., Ruangsittichai, J., Saralamba, N.T., Thaenkham, U., 2016. A molecular phylogeny of Asian species of the genus *Metagonimus* (Digenea) – small intestinal flukes – based on representative Japanese populations. Parasitol. Res. 115,1123–1130. <https://doi.org/10.1007/s00436-015-4843-y>.

Sato, H., Ihara, S., Inaba, O., Une, Y., 2010. Identification of *Euryhelmis costaricensis* metacercariae in the skin of Tohoku hynobiid salamanders (*Hynobius lichenatus*), northeastern Honshu, Japan. J. Wildl. Dis. 46,832–842. <https://doi.org/10.7589/0090-3558-46.3.832>.

Scholz, T., Kuchta, R., Barčák, D., Cech, G., Oros, M., 2024. Small intestinal flukes of the genus *Metagonimus* (Digenea: Heterophyidae) in Europe and the Middle East: A review of parasites with zoonotic potential. Parasite*.* 31,20. <https://doi.org/10.1051/parasite/2024016>.

Shumenko, P.G., Tatonova, Y.V., Besprozvannykh, V.V., 2017. *Metagonimus suifunensis* sp. n. (Trematoda: Heterophyidae) from the Russian Southern Far East: Morphology, life cycle, and molecular data. Parasitol. Int. 66,982–991. <https://doi.org/10.1016/j.parint.2016.11.002>.

Tatonova, Y.V., Shumenko, P.G., Besprozvannykh, V.V., 2013. Description of *Metagonimus pusillus* sp. nov. (Trematoda: Heterophyidae): phylogenetic relationships within the genus. J. Helminthol. 92,703–712. <https://doi.org/10.1017/s0022149x17001146>.

Vojtek, J., 1974. Metacercariae from fishes of Czechoslovakia. Folia Fac. Sci. Nat. Univ. Purkynianae Brun. 15,13–51.

Zdun, V.I., 1961. Larval trematodes in freshwater molluscs of the Ukraine. Publishing House of the Ukrainian Academy of Sciences, Kiev. (in Russian)
